# Genome-Wide Identification, Evolution and Expression Analysis of *mTERF* Gene Family in Maize

**DOI:** 10.1371/journal.pone.0094126

**Published:** 2014-04-09

**Authors:** Yanxin Zhao, Manjun Cai, Xiaobo Zhang, Yurong Li, Jianhua Zhang, Hailiang Zhao, Fei Kong, Yonglian Zheng, Fazhan Qiu

**Affiliations:** National Key Laboratory of Crop Genetic Improvement, Huazhong Agricultural University, Wuhan, China; Universidade Federal do Rio Grande do Sul, Brazil

## Abstract

Plant mitochondrial transcription termination factor (*mTERF*) genes comprise a large family with important roles in regulating organelle gene expression. In this study, a comprehensive database search yielded 31 potential *mTERF* genes in maize (*Zea mays* L.) and most of them were targeted to mitochondria or chloroplasts. Maize *mTERF* were divided into nine main groups based on phylogenetic analysis, and group IX represented the mitochondria and species-specific clade that diverged from other groups. Tandem and segmental duplication both contributed to the expansion of the *mTERF* gene family in the maize genome. Comprehensive expression analysis of these genes, using microarray data and RNA-seq data, revealed that these genes exhibit a variety of expression patterns. Environmental stimulus experiments revealed differential up or down-regulation expression of maize *mTERF* genes in seedlings exposed to light/dark, salts and plant hormones, respectively, suggesting various important roles of maize *mTERF* genes in light acclimation and stress-related responses. These results will be useful for elucidating the roles of *mTERF* genes in the growth, development and stress response of maize.

## Introduction

Mitochondria and chloroplasts possess their own genetic materials containing a few dozen genes required for gene expression, photosynthesis and the electron transport chain, since most genes of these organelles have been either lost or transferred to the nucleus during evolution from their bacterial progenitors in different plant species [1]. Most of approximately 2000 and 2600 proteins located in plant mitochondria and chloroplasts, respectively, are encoded in the nuclear genome [2]. Despite their small genomes, the mitochondria and plastids in higher plant have complex transcription machineries. Several components of the transcriptional machinery have been reported, such as nuclear-encoded phage-type RNA polymerases for mitochondria and chloroplasts, and plastid-encoded cyanobacterial-type RNA polymerases and nuclear-encoded sigma-like factors for chloroplasts; however, additional unidentified auxiliary factors are required for organellar transcription [3], [4].

Recently, *mTERF* homologs predicted to be imported in both chloroplasts and mitochondria were identified in *Arabidopsis thaliana* [5], [6] as putative organellar transcription factors, since mTERF is a transcription terminator in animal mitochondria. The mitochondrial Transcription tERmination Factor (*mTERF*) family consists of a group of proteins in human. These proteins have a modular architecture based on repetitions of a 32-amino acid (aa) mTERF motif which contains three leucine zipper-like elements [7], [8], and this has been suggested to be the basis of their ability to bind DNA [9]. The molecular function of *mTERF*s has so far only been described for metazoan members of the protein family. Vertebrates have four *mTERF* paralogs. In humans, mTERF1 is a sequence-specific DNA-binding protein responsible for mitochondrial transcription termination at the 3′-end of the 16S rRNA gene, promoting termination of transcripts from the first transcription initiation site (H1) [10], [11]. mTERF2 can bind to mitochondrial DNA [12] and, at least in mouse, seems to influence transcription [13]. mTERF3 acts as a specific repressor of mammalian mtDNA transcription initiation *in vivo* [14]. mTERF3 binds to the mtDNA promoter region, and depletion of mTERF3 increases transcription initiation on both mtDNA strands. mTERF4 can form a stoichiometric complex with the ribosomal RNA methyltransferase NSUN4 to directly control mitochondrial ribosomal biogenesis and translation [15], [16].

*mTERF* genes have been found in monocotyledonous and dicotyledonous nuclear genomes, in the moss *Physcomitrella patens*, but are apparently absent in fungi and prokaryotes [6], [17], [18]. Flowering plants have the highest number of *mTERF* genes among eukaryotes, and most annotated Arabidopsis *mTERF*s are targeted to mitochondria or chloroplasts [17], [18]. To date, only five *mTERF* genes from plants have been characterized: *MOC1* (*mterf-like gene of Chlamydomonas 1*) from *Chlamydomonas reinhardtti* [19], [20], and *SOLDAT10* (*SINGLET OXYGEN-LINKED DEATH ACTIVATOR 10*) [21], *BELAYA SMERT/RUGOSA2*(*BSM/RUG2*) [17], [22], *MDA1* (*MTERF DEFECTIVE IN Arabidopsis1*) [23] and *SHOT1* (*suppressor of hot1-4 1*) [24] from Arabidopsis.

In the unicellular green alga *C. reinhardtii*, MOC1 binds specifically to an octanucleotide sequence within the mitochondrial rRNA-coding module *S3*, and a loss of MOC1 increases read-through transcription at the S3-binding site thereby causing elevated antisense RNA levels in the mutant *stm6*, suggesting that *MOC1* possesses the evolutionarily-conserved transcription termination activity as for *mTERF1* in human [20]. *SOLDAT10*, the first *mTERF* gene characterized in higher plants, is localized to chloroplasts and its loss reduces plant growth and pigmentation while complete inactivation of *SOLDAT10* is apparently lethal. The *soldat10* mutant has decreased levels of plastid-specific rRNAs and affects protein synthesis in plastids, which subsequently activates retrograde signaling to the nucleus and leads to overexpression of stress-related nuclear genes [21]. Another Arabidopsis *mTERF*, dubbed *MDA1*, is targeted to chloroplast, and *mda1* mutant exhibit altered chloroplast morphology and plant growth. Additionally, the *mda1* mutations enhance salt and osmotic stress tolerance and alter sugar responses during seedling establishment via perturbing abscisci acid (ABA) retrograde signaling [23]. *BSM* or *RUG2*, encoding a dual-targeted mTERF protein, has a broader function, and is essential for normal plant development. Its loss affects levels of transcripts in both mitochondria and chloroplasts [17], [22]. *SHOT1* is characterized as one suppressor of Arabidopsis *hot1-4* mutant and resides in mitochondria. The *shot1* mutant can suppress the *hot1-4* heat-hypersensitive phenotype via changing mitochondrial function and increasing transcripts of other heat shock protein (HSP) genes. Expression alteration of other *HSPs* and redox-related genes in *shot1-2* are involved in retrograde signaling from mitochondria to nucleus [24]. Taken together, *mTERF* genes are required for organelle gene expression regulation and play important roles in plant growth, development and abiotic stress tolerance, at least in Arabidopsis and possibly other higher plants. However, little is known about the molecular mechanisms of *mTERF* that control transcription of the mitochondrial and chloroplastic genomes. More *mTERF* genes require characterization in diverse plants, especially crop plants.

In this report, 31 putative *mTERF* genes were identified in the maize genome. Most maize mTERF proteins are predicted to reside in mitochondria or plastids. Phylogenetic analysis of *mTERF* genes in maize, rice, and Arabidopsis indicates mitochondria- and plastid-targeting *mTERF* proteins form two divergent clades. Expression of *mTERF* genes are regulated in maize seedlings treated with light/dark, plant hormones and salts, showing their important roles in abiotic stress response. Our work will provide a basic biochemical characterization of maize *mTERF*s, paving the way for future functional studies and certainly contributing to improving knowledge of their role in plant biology.

## Results

### Identification and Nomenclature of *mTERF* Genes in Maize

Using keyword and homology searches, we obtained 90 maize mTERF proteins from the NCBI Protein Database, 26 maize *mTERF* unigenes from the NCBI Unigene Database, 26 *mTERF* cDNA sequences from the Maize Full Length cDNA Library Database, and 30 *mTERF* genes from maize genomes that were identical to maize *mTERF* genes identified by HMMER 3.0 [25] with mTERF PFAM file (PF02636) (Table S1). Sequence comparison for the above putative mTERF cDNAs and proteins indicated that all *mTERF* cDNA sequences and unigenes (except *Zm#S48278749*) were derived from 27 of 30 *mTERF* genes annotated in the maize genome and 87 of 90 maize mTERF proteins from GenBank were encoded by 26 of 30 *mTERF* genes identified in the maize genome; while the remaining three proteins and *Zm#S48278749* were assumed to be from two undiscovered *mTERF* genes in maize (Table S1). Two *mTERF* genes, *GRMZM2G426154* and *GRMZM2G175930*, were located in AC217966.3-Contig20 (forward strand) and AC205479.3-Contig45 (reverse strand), respectively, which were about 38 kb apart in the maize genome, and matched distinct parts of one *mTERF* cDNA (Figure S1). Therefore, *GRMZM2G426154* and *GRMZM2G175930* could be mis-annotated and could be two parts of one *mTERF* gene separated by unsequenced genomic gaps (Figure S1). Finally, 31 unique *mTERF* genes were identified in maize and designated *ZmTERF1*–*ZmTERF31* according to the order of these genes localizing in the chromosomes except for *ZmTERF30* and *-31* (Table S2); and *ZmTERF23* was the gene derived from *GRMZM2G426154* and *GRMZM2G175930* (Table 1). SMART [26] database searching revealed that each of the 31 maize *mTERF* gene products had at least one mTERF motif. *ZmTERF1*–*ZmTERF29* were selected for further study in this work since they had known genomic DNA sequences.

**Table 1:** Information of *mTERF* gene family in maize.

Gene Name^a^	Gene Locus^b^	Protein ID^c^	ORF(bp)^d^	Deduced protein^e^			Subcellular Location^f^	
				Length (aa)	MW(kDa)	pI	*in silico*	Proteomics†
ZmTERF1	GRMZM2G168665	DAA45475.1	912	303	32.53	10.44	C/C	C
ZmTERF2	GRMZM2G061542	DAA48102.1	1950	649	73.88	8.92	M/M	
ZmTERF3	GRMZM2G034217	ACG24064.1	1185	394	44.20	9.99	M/M	
ZmTERF4	GRMZM2G054517	DAA52215.1	651	216	24.24	9.78	−/−	
ZmTERF5	GRMZM2G159766	ACG24523.1	1203	400	43.67	10.00	M/M	
ZmTERF6	GRMZM2G170137	NP_001140442.1	645	214	23.65	8.93	−/C	C
ZmTERF7	GRMZM2G087679	This study	357	118	14.08	9.33	C/−	
ZmTERF8	GRMZM2G060114	ACG38629.1	1170	389	43.06	9.37	M/M	C
ZmTERF9	GRMZM2G130773	DAA41241.1	1527	508	57.95	8.98	M/M	C
ZmTERF10	GRMZM2G177019	NP_001144077.1	990	329	36.92	10.33	M/M	
ZmTERF11	GRMZM2G023257	NP_001151049.1	1167	388	42.98	9.93	M/M	
ZmTERF12	GRMZM2G113181	NP_001152615.1	1005	334	37.34	9.63	−/−	
ZmTERF13	GRMZM2G312806	NP_001141758.1	840	279	31.16	10.49	M/M	
ZmTERF14	GRMZM2G000610	NP_001145894.1	1470	489	54.68	8.09	M/M	
ZmTERF15	GRMZM2G119921	ACL52777.1	1188	395	44.62	9.30	M/M	
ZmTERF16	GRMZM2G395850	NP_001143033.1	1068	355	40.12	9.47	M/M	
ZmTERF17	GRMZM2G024550	ACF87053	1461	486	54.33	9.24	C/C	C
ZmTERF18	GRMZM2G017355	AFW73453.1	1176	391	42.83	9.31	M/C	C
ZmTERF19	GRMZM2G017429	NP_001169079.1	1173	390	42.43	9.11	ER/S	C
ZmTERF20	GRMZM2G158854	AFW73456.1	411	136	15.25	9.39	−/S	
ZmTERF21	GRMZM2G161146	DAA48102.1	1116	371	40.83	9.76	−/M	
ZmTERF22	GRMZM2G012999	AFW77957.1	1212	403	45.16	9.96	M/M	
ZmTERF23*	GRMZM2G426154	NP_001152167.1	1836	611	68.67	9.52	C/C	C
	GRMZM2G175930							
ZmTERF24	GRMZM2G142150	NP_001169565.1	1839	612	68.43	9.34	C/C	C
ZmTERF25	GRMZM2G062910	NP_001147866.1	1725	574	64.75	6.48	M/M	
ZmTERF26	GRMZM2G325350	NP_001130068.1	1155	384	42.90	9.47	M/M	
ZmTERF27	GRMZM2G029933	NP_001149660.1	1485	494	55.23	5.70	C/C	C
ZmTERF28	GRMZM2G068462	This study	510	169	18.58	9.94	−/−	
ZmTERF29	GRMZM2G157716	AFW88319.1	906	301	32.47	9.81	C/M	C
ZmTERF30		NP_001152154.1	900	290	33.25	9.81	ER/C	
ZmTERF31		ACG24302.1	999	332	37.71	9.12	−/−	

a Systematic nomenclature of maize mTERF genes according to the order of their location in maize chromosomes.

b Gene name annotated in MaizeGDB (http://www.maizegdb.org).

c NCBI accession number of the maize mTERF proteins; -, no identical protein found in NCBI Protein Database.

d Length of open reading frame (ORF) of maize *mTERF* genes in base pairs.

e Length (number of amino acids, aa), molecular weight(MW) (kilodaltons, kDa) and isoelectric point (pI) of the deduced proteins.

f Subcellular location of maize mTERF proteins predicted by Predotar [28] and (/) TargetP [29]. C, Chloroplast; M, Mitochondria; S, Secretion; ER, Endoplasmic Reticulum; -, none.

† The ZmTERF proteins were identified in maize plastid nucleoid or stroma in previous proteomic studies [31], [32].

*Gene model of the maize *mTERF*s repredicted in this study instead of those annotated in MaizeGDB (http://www. maizegdb.org).

Our results showed that the maize, rice and Arabidopsis genomes encoded a similar number of mTERF proteins, which comprises 34 members identified in rice (*Oryza sativa* subsp. japonica) and 35 members in Arabidopsis [17], [18]. RT-PCR assay was carried out to validate cDNA sequences of the *mTERF* genes with gene-specific primers listed in Table S3. Except for *ZmTERF4*, *-7* and *-20*, which had undetectable expression in B73 seedling leaves, most cDNA sequences of the 29 maize *mTERF* genes were obtained (Figure S2) and were identical to corresponding gene models annotated in MaizeGDB (http://www.maizegdb.org/). All identified *mTERF* genes encode proteins varying from 118 (ZmTERF7) to 649 aa (ZmTERF2), in which protein sizes of ZmTERF4, -7 and -20 were <150 aa (Table 1). Isoelectric points of most maize mTERF proteins were similar and >8.0, except for those of ZmTERF25 (6.48) and ZmTERF27 (5.70) (Table 1).

### Phylogenetic Analysis and Classification of *ZmTERF* Genes

**Figure 1:**
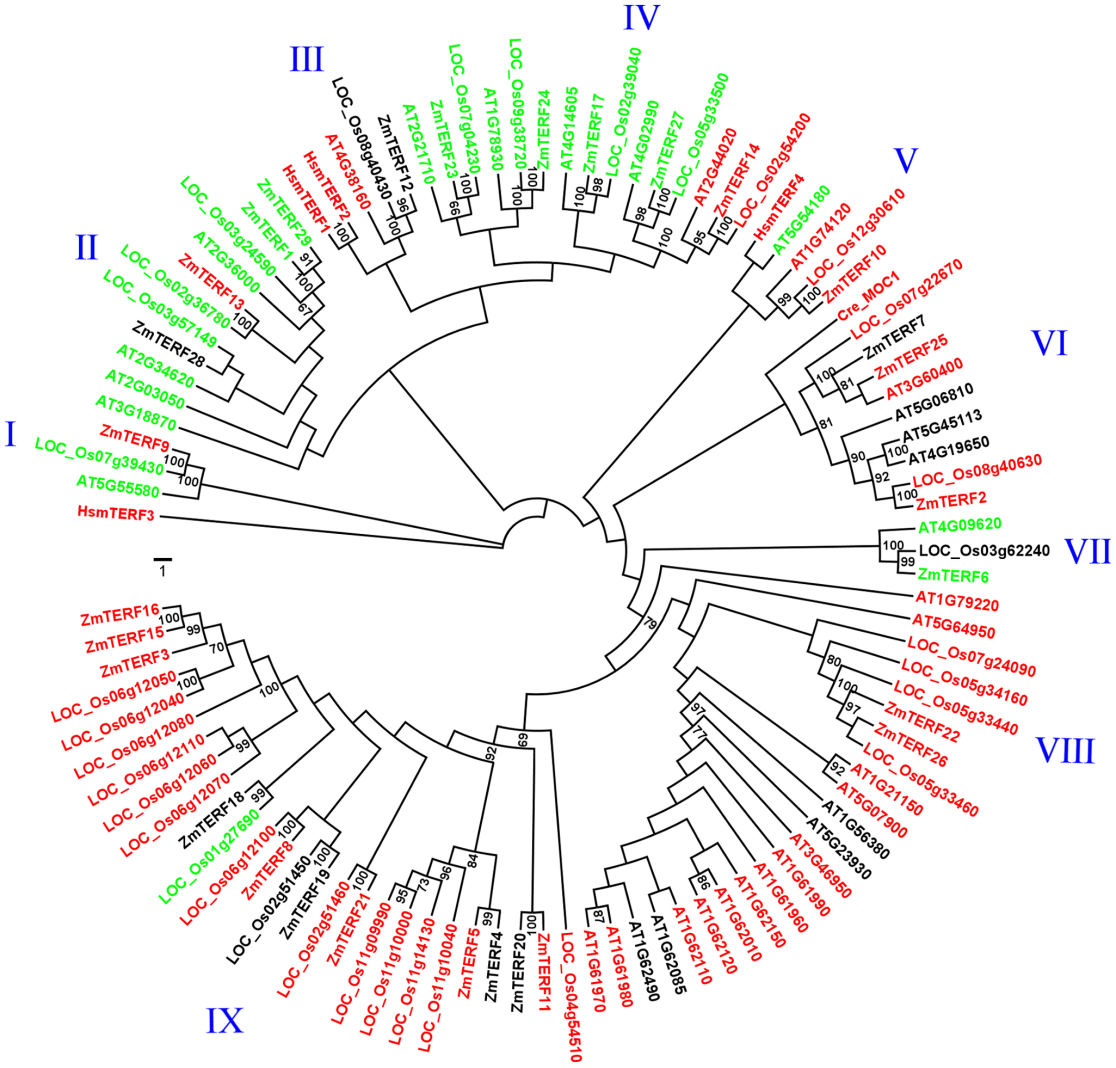
Phylogenetic analysis of maize and other known mTERF proteins. Phylogeny tree was constructed using RAxML with Arabidopsis, maize and rice mTERF protein sequences. The numbers associated with the branches indicate the bootstrap values (%) for 500 bootstrap replicates and only bootstrap values of >60% are shown. The scale bar represents the number of amino acid substitutions per site. Nine groups designated I–IX are shown outside. Subcellular localization of mTERF proteins is shown in red for mitochondria, in green for chloroplasts and in black for other places or a discrepancy in prediction by TargetP [28] and Predotar [29].

To evaluate the evolutionary relationships among the *mTERF* genes in maize, rice and Arabidopsis, we performed a phylogenetic analysis of the 98 mTERF protein sequences to construct an unrooted ML inference-based tree with RAxML [27]. The defined multiple alignment sequences for phylogenetic tree construction are found in File S1. The phylogenetic tree also included known *mTERF* genes identified in human and *C. reinhardtii*. The best ML scoring tree is shown in Figure 1. From the tree topology resembling that from Arabidopsis phylogenetic trees constructed previously [17] including lower plants, it was apparent that there were nine groups (groups I–IX) despite the low bootstrap values for deep nodes (Figure 1). Group IX contained 11 *mTERF* maize genes while the other groups had 1–5 members. It is noteworthy that all functionally identified Arabidopsis *mTERF* genes belonged to groups II, IV and VI with *SOLDAT10* (AT2G03050) in group II, *MDA1* (AT4G14605) and *BSM/RUG2* (AT4G02990) both in group IV, and *SHOT1* (AT3G60400) in group VI.

Relationship analysis of intragroup members with well-supported bootstrap values revealed that seven groups (group I–VII) included both proteins from eudicots and monocots (Figure 1). This suggested that nearly 80% of the groups here defined were already present in the common ancestor of both groups, and evolved before divergence of monocots and dicots. Therefore, these *mTERF* genes might play conservatively important roles in mediating expression of organellar genes as do corresponding characterized Arabidopsis *mTERF* genes. Group IX comprised monocot-specific genes and its members were of similar protein length, except for ZmTERF4 and ZmTERF20. Another clade containing exclusive Arabidopsis mTERF proteins was found in the evolutionary tree and diverged from group VIII *mTERF* genes. This result suggests that group IX formed after divergence of monocots and dicots, and expansion of subgroups of group IX in monocots took place before divergence of rice and maize. However, we cannot exclude the possibility that rice-specific subgroups in group IX evolved after species formation of rice (Figure 1), because syntenic orthologs of several maize *mTERF* genes were not be found in rice genome (Table S2). Putative paralogous genes were identified with the criterion that two mTERF proteins were clustered pairwisely and supported by >95% bootstrap values, leading to formation of three pairs of maize *mTERF* paralogs, including *ZmTERF15* vs. *ZmTERF16*, *ZmTERF4* vs. *ZmTERF5*, and *ZmTERF11* vs. *ZmTERF20*.

### Prediction of Potential Subcellular Location for ZmTERF Proteins

Unlike human mTERF proteins which all reside in mitochondria, GFP fusion transient expression experiments showed that most members of Arabidopsis mTERF proteins were targeted to mitochondria or chloroplasts [17]. To investigate the subcellular location of ZmTERF proteins, we performed *in silico* prediction using TargetP [28] and Predotar [29]. Totally, 25 of 31 ZmTERF proteins were predicted to enter mitochondria or chloroplast in either TargetP or Predotar (Table 1), and three mTERF proteins ZmTERF19, -20 and -30 could be secretory proteins, while four mTERF proteins (ZmTERF4, -12, -28 and -31) were predicted to localize to any other location (Table 1). In this result, six maize mTERF proteins (ZmTERF6, -7, -18, -20, -21, and -29) were targeted to different organelles predicted by the two programs. In the six proteins, ZmTERF18 and ZmTERF29 proteins possibly have dual transit peptides for mitochondria and chloroplasts.

Mitochondrial and plastid proteomic database identification provide another way to survey the compartmentalization of the proteins of interest. Here we used the maize mTERF proteins to screen PPDB [30], and found that ten mTERF proteins (ZmTERF1, -8, -9, -17, -18, -19, -23, -24, -27 and -29) were detected in maize plastid nucleoid [31] and ZmTERF6 was observed in maize chloroplast stroma (Table 1 and Table S2) [32]. Of these mTERF proteins, ZmTERF8 and ZmTERF9 are not consistent with the prediction produced by TargetP [28] and Predotar [29] (Table 1). Subcellular targeting of rice mTERF proteins were also predicted using TargetP [28] and Predotar [29] (Table S3). By mapping the subcellular location information of plant mTERF proteins on the evolutionary tree (Figure 1), we found that the *mTERF* genes can group according to targeting predictions in the evolutionary tree (Figure 1). Intragroup mTERF proteins localize to the same organelles with several exceptions, such as ZmTERF9 in group I, ZmTERF13 in group II, and LOC_Os01g27690 in group IX. Groups I, II, IV and VII targeted to the chloroplast, and groups III, V, VI, VIII and IX targeted to the mitochondria. Surprisingly, there was a mitochondria-targeted subgroup in group IV, including AT2G44020, ZmTERF14 and LOC_Os02g54200.

### Gene Structure and Conserved Motifs of *ZmTERF* Genes

Studying the structure of genes of interest is crucial in biology and can provide important clues concerning gene evolution. The exon–intron organization of maize *mTERF* genes was obtained by comparing the cDNA sequences of maize *mTERF* genes with the corresponding genomic DNA sequences. The maize *mTERF* cDNA sequences were derived from sequenced PCR products (Figure S2), which were amplified from B73 seedling leaf total RNA with gene-specific primers (Table S4) except for three genes *ZmTERF4*, *-7* and *-20* which were not detected in this study and of which cDNA sequences were extracted from corresponding gene models annotated in MaizeGDB (http://www.maizegdb.org/). The genomic DNA sequences were retrieved from MaizeGDB (http://www.maizegdb.org/). This result showed that quite a large number of *mTERF* genes (79%) were intron-free (Figure S3). Six *mTERF* genes containing introns belonged to groups I, III, IV and VII, of which only group IV had multiple members. *ZmTERF12* (group III) had a 5′-UE (Untranslated Exon) in 5′-UTR (UnTranslated Region) and had no intron in the coding sequence, while *ZmTERF6* (group VII) had multiple 5′-UEs in 5′-UTR and only one intron in the coding sequence. There were two different transcripts for *ZmTERF6*, nevertheless, the alternative splicing event occurs in 5′-UTR and had no effect on the coding sequence (Figure S3). Most intron-free *mTERF* groups contained multiple members, particularly group IX, implying that ancestors of these groups were evolved via retrotransposition.

**Figure 2:**
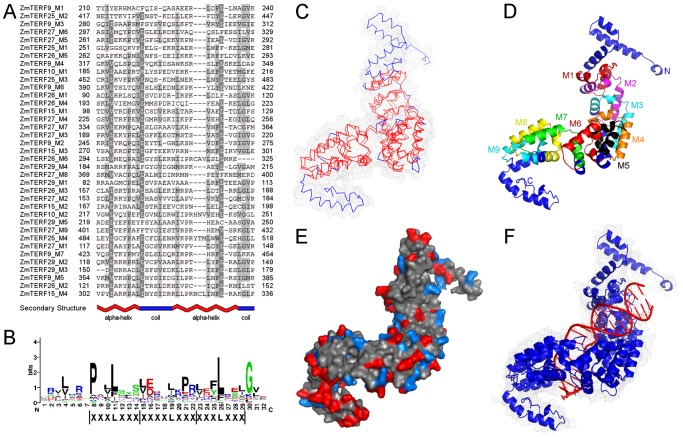
Structure of maize mTERF protein. (A) Multiple sequence alignment of maize mTERF motifs. Alignment of the conserved maize mTERF motifs was conducted with CLUSTALW2.0, and displayed using GeneDoc with the conserved residue shading mode. The conserved secondary structure was predicted by MINNOU [69] and shown under the alignment panel. (B) Sequence representation LOGO derived from multiple sequence alignment of the mTERF motifs and three leucine zipper-like X_3_LX_3_ repeats are highlighted. (C) Overlay between human mTERF1 model (PDB 3MVA) and ZmTERF27 structure developed by I-TASSER [42]. The 3D structures of HsmTERF1 and ZmTERF27 proteins are indicated by blue and red stricks, respectively. The matched regions are represented by double red stricks. (D) ZmTERF27 model is displayed in cartoon and nine mTERF motifs are indicated in different colors. (E) Electrostatic surface potential of ZmTERF27. Only basic amino acid residues (histidine, arginine and lysine) and acidic residues (aspartic and glutamic acids) were analyzed and are shown on the surface of ZmTERF27 model in light blue and red, respectively. (F) DNA-binding mode of ZmTERF27. The dsDNA (red) derived from human mitochondrial DNA was predicted to be docked in the groove formed by ZmTERF27 model (blue) using TM-align program [43].

Conserved mTERF motifs containing 32 aa residues have been characterized in human mTERF proteins and are thought to be DNA-binding modules [8]. To identify any possible known motifs in maize mTERF proteins, we carried out SMART and PFAM database searches and revealed that, except for mTERF motifs (approximately 38-aa SM00733 in SMART and 345-aa PF02536 in PFAM), no other known motifs were identified (Figure S4). This result suggested that these genes play conserved biological roles. For SM00733 mTERF motifs, maize mTERF proteins had 1–11 mTERF motifs. The intragroup members had similar protein architecture, for example group IX mTERF proteins had 4–6 mTERF motifs except for two putative pseudogenes, *ZmTERF7* and *ZmTERF20* (Figure S4), and group IV proteins with 9–11 mTERF motifs. To determine sequence features of maize mTERF motifs, 37 motif sequences within seven mTERF proteins from different groups were aligned with ClustalW 2.0 [33] and mTERF motif aa residues were graphically represented (Figure 2A). Three repeats of leucine zipper-like heptad X_3_LX_3_ identified in human mTERF proteins previously were also observed in maize mTERF motifs (Figure 2B). These results indicate that maize mTERF proteins harbor conserved mTERF motifs and these motifs contain conserved leucine resides like those in human mTERF motifs (Figure 2B) [8], implying plant mTERF proteins might possess similar structures and functions to human proteins.

**Figure 3:**
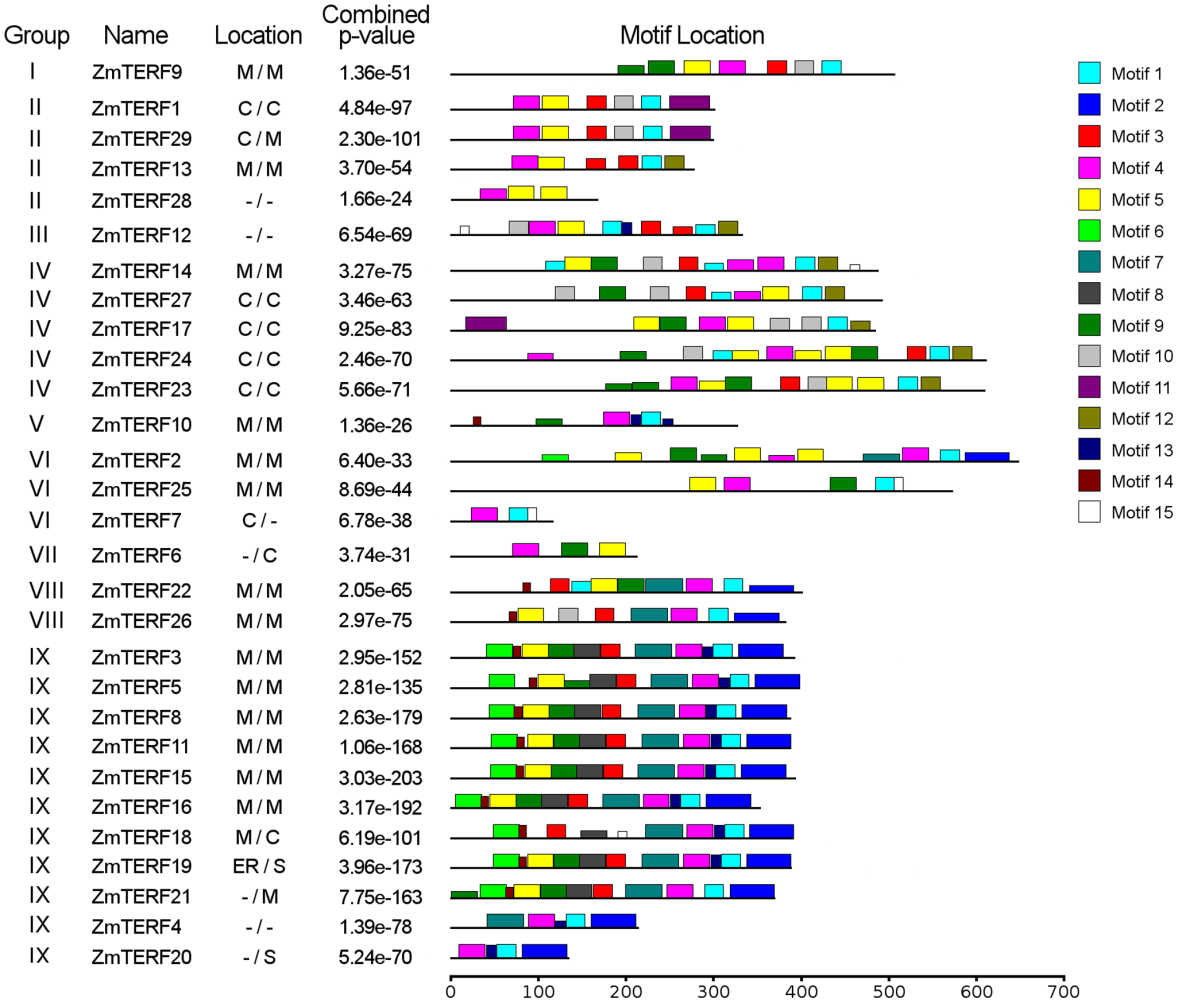
Schematic representation of putative conserved motifs identified MEME in maize mTERF proteins. Putative conserved motifs shared by maize mTERF proteins were mined in MEME program. Fifteen motifs are indicated by different colored boxes and the regular motif sequences are shown in the Table 2.

To discover putative motifs shared among related proteins within the maize *mTERF* family, the MEME program [34] was used. Overall, 15 highly significant motifs (<1e-50) were mined among these 29 proteins, and designated as motifs 1–15 (Figure 3). Of these motifs, motifs 1, 3, 4 and 5 were common to all maize mTERF proteins. Sequence comparison with PFAM mTERF seed alignment indicated that motifs 1, 3, 4, 5, 9, 10 and 12 overlaid with the PFAM mTERF domain (PF02536) (Table 2). Motifs 2, 6, 7, 8, 13 and 14 were specific to mitochondria-targeting mTERF proteins, especially to the group IX mTERF proteins (Figure 3). It is possible that group-specific motifs contribute to distinct functions of these *mTERF* genes.

**Table 2:** Multiple Em for Motif Elucidation (MEME) protein motifs detected in maize mTERF proteins.

Motif	Protein sequence (regular expression)*
1	[YE][LI][VA]E[FR]PA[LYF][LF]S[YF]SL[ED]RR[IL][VK]PR[HY]
2	**KGL[LV]NS[DN]RSF[FLY][STN]VAA[IL][GST][ED][ES]KFRS[KR][FY][VI]** **[HD]P[HY][KE][ED]SVP[GS]LA[AD]AYA[SA][AS]CAG[KE][VH][PQ]W**
3	[LI]LSY[DS][LIV]EKV[LV][KR]PN[LI][EA][FL]L[RQ][SE][CL]G
4	[ST]LG[VW]SEA[DE][VL]AR[AV]V[KS][KR]xPQ[ILV]LGFSEER[LIV]x[RK]
5	[FL]L[AS]ELG[LV]Sx[AK][ED][IL][AG][AK][VL][VLI][AR]RDPR[LV]LC[SA][SD][VI][DE]R
6	**F[AS][VA]EDYLVA[TAR]C[GH]LT[PQR][AE]QA[LA][KR][AV][SA]KH[LV]SHLK**
7	**P[ED][RS][VIL][QR]EA[LV]AR[AL]EE[FL]G[VIL][PQ][PR]G[ST][GQ][LM]** **FRH[AG][LV]x[ATV][VF]A[CIS]L[GS][PR]EK[IV]A[AS]K**
8	**R[HR][PS]S[FLV][VR][SL][KN][LV][EQ]F[WY][LI][SP][FLV][FL]GS[FLP][DE][RE][LV][LI][RQ]A[LV][RK]IN**
9	LAPR[VL]AEL[RL][DS]LG[LVF]SPSQ[IL]PR[LI]LxVxPxL[FL]
10	[ART][RKS][FY]PA[LV][LF][GT][LYC][SG][VL][DE][GK][NHK][LMI][RKV]P[KV][VAY][DEQ][YF]
11	P[LR][PG][AL][MQ]L[RP]P[GW][DER][AL][KS][FL][RS][ARS][TS][LS][DST]SC[VR][GS][SC][TM]L[PV][RS]R[RQ][SL][AP][LI][WC][HN]A[TQ][WS][VY][DA]DD[AL][ATW][AV]A
12	R[GN]Ix[CM]SLx[EW]ML[NT]C[NS]DEKFAER
13	**EFLIN[EV]VGLE[PV]**
14	**[SD][PA]S[KN][AP]DAV**
15	TML[RW]WLQEHG

*Mitochondrial mTERF protein-specific motifs are shown in bold, and the residues underlined overlap with the PFAM mTERF domain (PF02536).

### Higher Structure Analysis of ZmTERF Proteins

Human mTERF proteins regulate mitochondrial gene expression via binding to mitochondrial DNA (hsmTERF1–hsmTERF3) or RNA (hsmTERF4) [13]–[15], [35]. The structure of three of four mTERF proteins have been determined by X-ray diffraction in human [5], [16], [36]. The mTERF protein structure shows they are modular proteins, and the mTERF module comprises two or three tandem α-helixes [36]–[38]. There are eight modules in hsmTERF1 [37], seven in hsmTERF3 [36] and six in hsmTERF4 [38]. Human mTERF1 adopts a fold similar to HEAT [39], [40] and PUM/PUF proteins [41] and forms a positively charged groove in the protein surface where dsDNA is docked [36], [37]. The overall structure of mTERF proteins are very different from previously proposed models [8], [11]; however, no structure has been resolved for plant mTERF proteins yet. Arabidopsis BSM/RUG2 protein was shown to bind to dsDNA in a nonspecific manner, and the structure of the protein was modeled based on homology to human mTERF1 [17].

Here, the structure of ZmTERF27 protein without N-terminal transit peptide, a maize mTERF protein homologous to hsmTERF1 and BSM/RUG2, was developed using I-TASSER program [42]. The 3D structure modeling of maize mTERF protein (Figure 2C) was similar to the structure of hsmTERF1. ZmTERF27 contains nine mTERF modules which differ from the mTERF motifs mined by SMART [26] (Figure 2D and Figure S5). ZmTERF27 protein structure can fold to form a positively charged groove (Figure 2E), suggesting that it could bind to dsDNA as does human mTERF1, and the 3D structure complex including ZmTERF27 and human mitochondrial DNA was developed using the TM-align program [43]. This result indicates that plant *mTERF* genes, or at least parts of them, play conserved roles in regulating organellar gene expression by DNA-binding activity similar to human *mTERF* genes. Compared with hsmTERF1, however, ZmTERF27 contained two excess domains in N- and C-termini, respectively (Figure 2). Even in the homologous region, the mTERF modules of ZmTERF27 cannot match those modules in hsmTERF1 very well due to unaligned aa residues (Figure 2 and Figure S5). These variations demonstrate that ZmTERF27 has specialized biological function in plants. Other maize mTERF proteins containing >4 mTERF motifs can also be modeled to form 3D structures like that of ZmTERF27 except for ZmTERF2 and -25 (data not shown).

### Chromosomal Location and Gene Duplication Analysis of *ZmTERFs*

**Figure 4:**
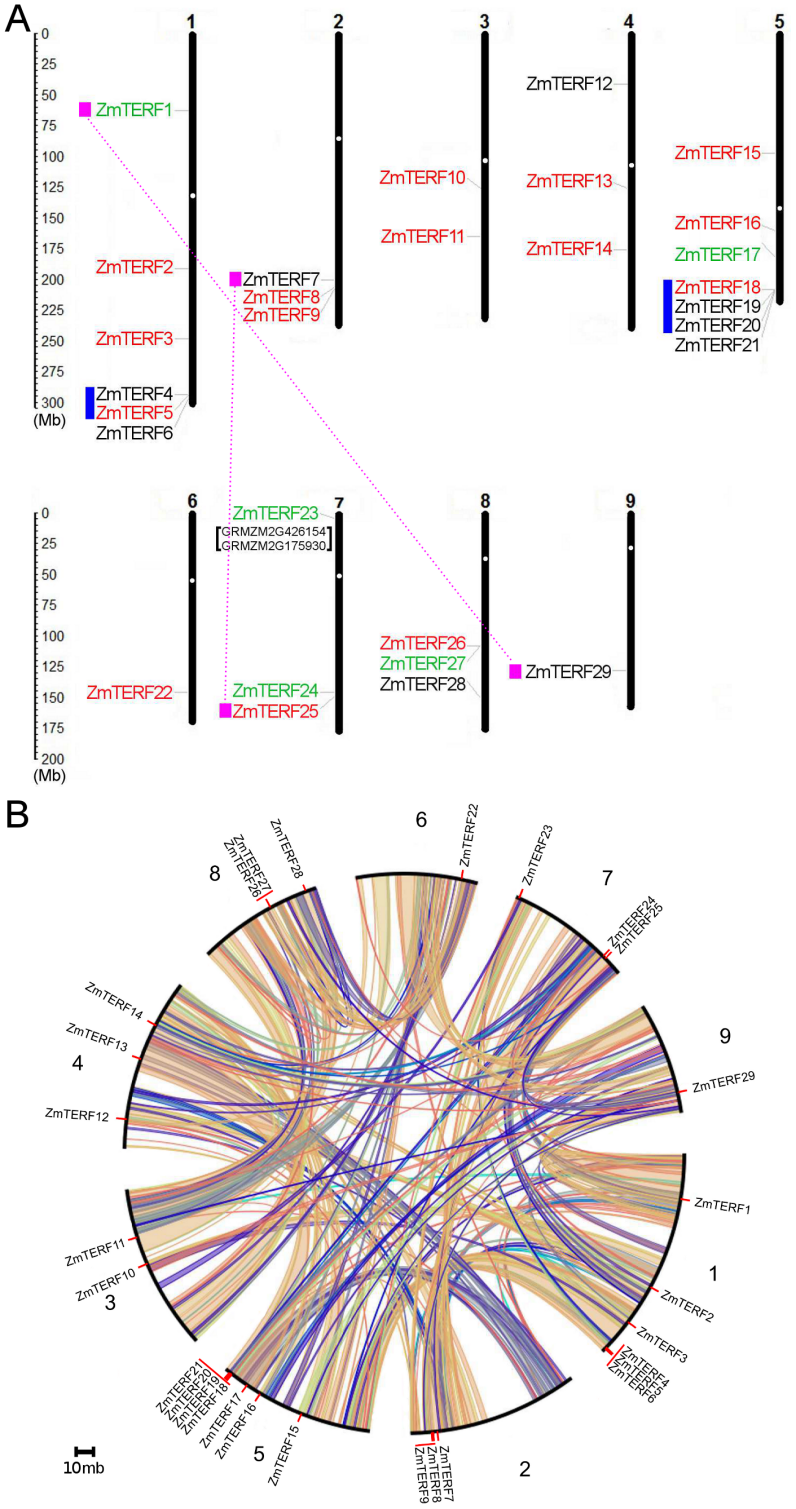
Chromosomal localization and gene duplication events of maize *mTERF* genes. (A) Distribution of *ZmTERF* genes in maize chromosomes. Twenty-nine *ZmTERF* genes were mapped on the nine maize chromosomes. Segmentally duplicated paralogs are connected by dotted magenta line, and tandemly duplicated homologs are marked by blue bars before the genes. The centromere of each chromosome is indicated with a white circle, and chromosome numbers are indicated at the top of each chromosome. (B) Duplicated blocks in maize chromosomes. The circular image retrieved from PLAZA database [45] show inter-chromosome homologous regions connected by bands in different colors. The chromosome numbers and *mTER*F genes are indicated outside.

To investigate the genomic organization of *ZmTERF* genes, the positions of the *mTERF* loci on maize chromosomes were mapped. The distribution of 29 *ZmTERF* genes on nine of ten maize chromosomes was uneven (Figure 4). Chromosomes 1 and 5 have six and seven *ZmTERF* genes and only one *mTERF* gene was observed in chromosomes 6 and 9, respectively. Analysis of maize *mTERF* genes in SyMAP [44] and PLAZA [45] revealed four (i.e. two pairs) *mTERF* genes could be assigned to maize segmental duplication and five *mTERF* genes could be involved in tandem duplication (Figure 4). The five tandemly duplicated *mTERF* genes were localized on chromosomes 1 and 5, respectively, and fell into two clusters, one for *ZmTERF4* and *ZmTERF5* and the other for *ZmTERF18*, -*19* and -*20* (Figure 4). The duplicated genes were inconsistent with those identified in the phylogenetic tree because two *ZmTERF* genes (*ZmTERF11* and *ZmTERF20*) were not in homologous regions. All of these duplicated genes exhibited high sequence similarity, especially *ZmTERF15* and *ZmTERF16* which shared a 343-aa peptide. Three pairs of homologs involving two tandem duplication events and one segmental duplication event belonged to group IX, while two other pairs were of groups II and IV, respectively. It is worth noting that in three pairs of duplicated *mTERF* genes, *ZmTERF4*, *-7* and *-20* genes encoding truncated redundant peptides were silent in maize compared with their corresponding paralogous copies. These findings suggest tandem and segmental duplication events both contributed to the current complexes of the maize *mTERF* gene family unlike Arabidopsis *mTERF* genes which were duplicated mainly by tandem duplication events [17], [18].

### Analysis of *cis*-elements in Promoters of *ZmTERF*s

**Figure 5:**
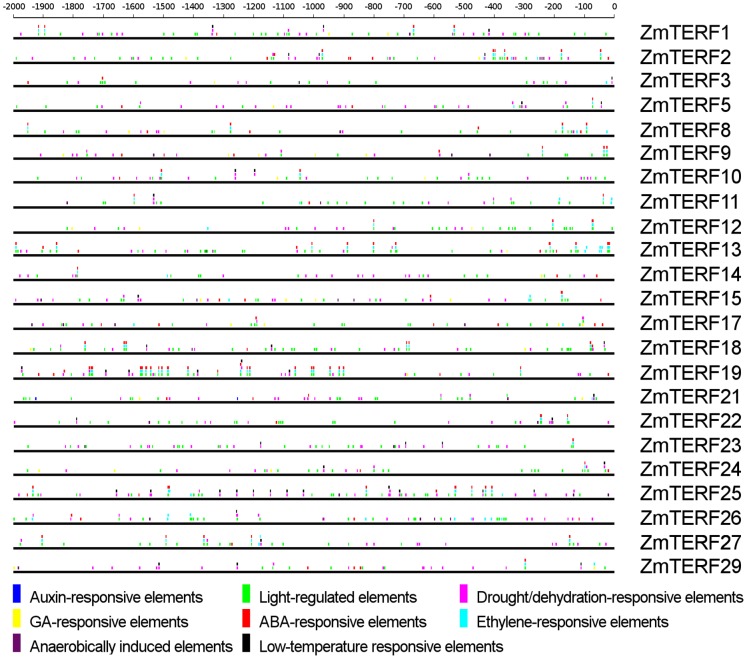
Phytohormone- and abiotic stress-responsive *cis*-elements in the promoter regions of maize *mTERF* genes. Putative LTRE, DRE, ARE, LRE, GARE, ABRE, ERE, AIRE and LTRE core sequences in the 2000-bp promoter regions of maize *mTERF* genes were predicted using PlantPAN [49]. The lines denote promoter sequences. Only the elements represented by different color boxes and located in the forward strand (sense strand of the gene) are indicated above the lines.

The *cis*-elements are important molecular switches involved in the transcriptional regulation of genes during plant growth and development, and abiotic stress responses [46]–[48]. The 2-kb promoter sequences upstream from the translation start codon of 23 maize *mTERF* genes were retrieved from maize genome sequences in MaizeSequence (http://www.maizesequence.org/index.html) and used for stress-related *cis*-element analysis in the PlantPAN server [49]. All of putative environment stimulus-responsive *cis*-elements were identified in *mTERF* gene promoters and divided into eight classes: including Auxin-responsive element (ARE), Light-regulated element (LRE), Drought/dehydration-responsive element (DRE), Gibberellic acid (GA) responsive element (GARE), ABA-responsive element (ABRE), Ethylene-responsive element (ERE), Anaerobically induced element (AIE) and Low-temperature responsive element (LTRE). Some *cis*-elements may respond to multiple plant hormones or environment stimuli – for instance LTRECOREATCOR15 (CCGAC) is induced by ABA, drought and low-temperature [48]. Only the *cis*-elements in positive strands were collected and displayed in Figure 5. The detailed information of stress-related *cis*-element sequences and annotation is found in Table S5, and the position and abundance of all *cis*-elements predicted to localize in promoter regions of maize *mTERF* genes are shown in Table S6.

All 23 *mTERF* promoters were highly enriched with LREs, and the average number of the LRE sequences in these promoters was 19.5, of which only five genes had <15 LRE boxes (Figure 5). Of 23 *mTERF* promoters, 17 were rich in DREs with >10 DREs per promoter, in which *ZmTERF25* had 25 DREs. There were nine *mTERF* promoters containing >5 ABREs per promoter – especially *ZmTERF19*, *-2* and *-3*, with 25, 14 and 14 ABREs, respectively. We also found that many EREs appeared in maize *mTERF* promoters, and eight *mTERF* promoters each contained >5 EREs. In addition, *ZmTERF25* and *-19* contained 12 and six LTREs, respectively, in their promoters, and others had <4. The two *mTERF* genes also possessed multiple abiotic stress-responsive elements. There were 19 cold-, drought- and ABA-regulated elements in *ZmTERF25*; and 11 drought-, high-salt- and cold-responsive elements in *ZmTERF19* (Figure 5). Notably, six *mTERF* genes had 3–5 AIEs, suggesting these genes were probably induced by anaerobic conditions, such as waterlogging stress. Therefore, expression of maize *mTERF* genes would be regulated by various environmental factors.

### Expression Pattern Analysis of Maize *mTERF* Genes in Different Tissues

The gene expression patterns can provide important clues for the gene function. Two approaches were applied to analyze the expression patterns of the maize *mTERF* genes in different tissues and organs, including expressed sequence tag (EST) profiles and microarray data.

For analysis of EST profiles, the maize ESTdb in NCBI (http://www.ncbi.nlm.nih.gov/dbEST/) was searched using BLASTN program. Most maize *mTERF* genes were expressed – except for *ZmTERF4*, *-7* and *-20* with no EST sequence detected (Table S7), providing strong indication for expression of most *mTERF* genes; However, the frequency and source of ESTs for *mTERF* genes varied, indicating their differential expression patterns. The EST sequences for *mTERF* genes were mainly from six single different tissues (root, shoot, ear embryo, endosperm and tassel) and several cDNA libraries derived from multiple mixed tissues. *ZmTERF6* and *-8* showed high expression in all tissues and organs used here, and several maize *mTERF* genes have tissue-specific expression patterns: *ZmTERF1* in embryo, and *ZmTERF11*, *-21* and *-29* in shoot. Additionally, EST hits for *ZmTERF13*, *-18*, *-19*, *-23* and *-28* were only from mixed tissues.

**Figure 6:**
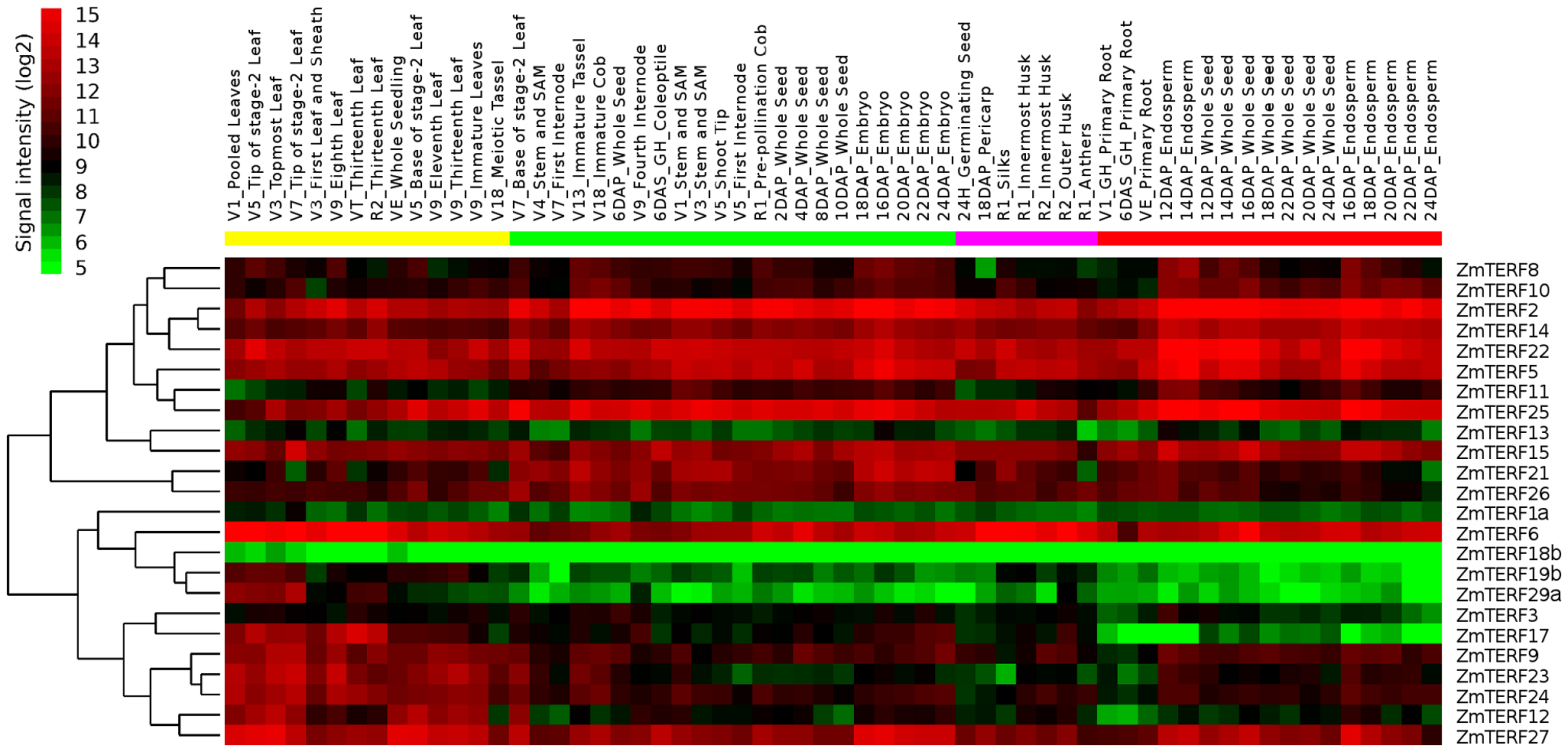
The expression profile of *mTERF* gene family in different tissues of maize. Dynamic expression profiles of maize mTERF genes in maize different tissues were derived from Nimblegen Maize Microarray data deposited in PLEXdb [72]. Relative expression level is denoted by intensity of red color (higher) or green color (lower). Two pairs of duplicated paralogs are marked by lowercase letters.

Expression patterns of maize *mTERF* genes were examined in different organs by estimating their transcript levels determined by Nimblegen maize microarray experiments encompassing 60 tissues at different developmental stages of maize: including germinating seed, primary root, whole seedling, stem and shoot apical meristem, internodes, cob, tassel and anthers, silk, leaf, husk and seed [50]. The detailed information for the tissues and organs was described by Sekhon *et al* [50]. The expression data for only 24 maize *mTERF* genes were collected, excluding *ZmTERF4*, *-7*, *-16*, *-20*, and *-28*. The signal values for all these maize *mTERF* genes are given in Table S8. The hierarchical clustering of microarray expression data using Cluster 3.0 [51] revealed expression patterns for 24 maize *mTERF* genes in diverse tissues and organs covering the whole life of maize, but no clearly distinct expression pattern groups emerged (Figure 6). The coefficient of variation (CV = sd/mean, where sd and mean represent the standard deviation and mean expression level of a gene across all the tissues, respectively) value of each *mTERF* gene was calculated to estimate the expression variation. This result showed that expressions of 18 *mTERF* genes were relatively stable across all tissues (CV<10%) (Table S8), implying that these genes were likely involved in basal metabolic or ‘housekeeping’ functions. In these genes, *ZmTERF2*, *-5*, *-6*, *-14*, *-15*, *-22*, *-25*, *-26* and *-27* were highly expressed throughout the 60-tissue panel and *ZmTERF1*, *-3* and *-13* genes were less expressed (Figure 6). The remaining eight stably expressed genes showed slight expression variation in certain tissues. *ZmTERF8* and *-10* showed similar expression patterns, with low expression in anther, root, pericarp and several types of leaves (Figure 6). *ZmTERF9* showed low expression in root and silk compared to other tissues. *ZmTERF24* was relatively less expressed in root, pericarp and germinating seed. *ZmTERF11* showed low expression in pericarp, silk, germinating seed and most types of leaves. The CV values of the other six genes (*ZmTERF12*, *-17*, *-18*, *-19*, *-23* and *-29*) were >10%, in which *ZmTERF17* and *-29* showed large expression variation with CV value >15%. The expression of *ZmTERF19* and *-29* was specific to leaf. *ZmTERF12* and *-17* were remarkably expressed in leaf and moderately expressed in embryo. The expression pattern of *ZmTERF23* is similar to that of *ZmTERF24*, and its expression level in leaf, endosperm, immature tassel and cob was higher than in other tissues or organs. Notably, *ZmTERF18* was lowest expressed over the whole life of maize plants. The chloroplast-targeting *mTERF* proteins, *ZmTERF6*, *-9*, *-23*, *-24*, *-27* and *-29* were highly expressed in leaves, especially *ZmTERF29* with leaf-specific expression, suggesting that these genes played important roles in chloroplast development and biogenesis.

To confirm the expression variations of maize *mTERF* genes, qRT-PCR was performed to measure the expression of eight *mTERF* genes in five different tissues, including mature seed, immature tassel, unpollinated silk, seedling leaf and root. In the eight genes, only *ZmTERF11* was targeted to mitochondria while the rest were all imported to chloroplasts. Of the senven chloroplast *ZmTERFs*, four *mTERF* genes (*ZmTERF19*, *-23*, *-27* and *-29*) exhibited similar expression patterns in the five tissues, and they were expressed predominantly in leaf. For other four mTERF genes, *ZmTERF6* was highly expressed in seed and leaf; *ZmTERF9* and *-24* showed high expression levels in seed, silk and leaf; and *ZmTERF11* was abundantly expressed in root (Figure S6). Additionally, all of the selected *ZmTERFs* showed lower expression levels in tassel compared to other tissues. These results were consistent with the observation in macroarray data.

Expression pattern shifts of the duplicated paralogous genes can reflect the maintenance of duplicate genes through nonfunctionalization, subfunctionalization or neofunctionalization [52]. For duplicated *mTERF* genes, two pairs (segmentally duplicated *ZmTERF1* and *ZmTERF29*, and tandemly duplicated *ZmTERF18* and *ZmTERF19*) both showed divergent expression profiles for duplicate genes. *ZmTERF1* and *ZmTERF18* showed low expression levels in all detected tissues or organs; whereas their paralogs, *ZmTERF29* and *ZmTERF19*, were preferentially expressed in leaf. This result indicates the fate of two *ZmTERF* pairs could be described as neofunctionalization since the expression of one copy of the paralog had an obvious increase in leaf (Figure 6).

### Expression Analysis of Maize *mTERF* Genes During Leaf Development

Corn is an important cereal and a typical C_4_ plant. In addition, plant *mTERF* genes can regulate chloroplast gene expression. It is speculated that maize *mTERF* genes may be involved in chloroplast biogenesis and development, and C_4_ photosynthesis occurring in the chloroplast. To validate this hypothesis, expression levels of maize *mTERF* genes during the development of C_4_ photosynthesis were extracted from RNA-seq data deposited in the eFP browser [53]. The RNA-seq data were derived from a leaf continuous developmental gradient and mature bundle sheath and mesophyll cells [54]. The leaf gradient comprises four representative zones of the leaf blade, including a basal zone (Base: sink tissue where cell division is active, cell-fates are being determined and proplastid appears), a transitional zone (Ligule+4: undergoing the sink–source transition), a maturing zone (Ligule+9: with strong light-mediated development and formation of mature chloroplast) and a mature zone (Tip-1: fully differentiated and active C_4_ photosynthetic zone) [54]. RPKM (reads per kilobase per million mapped reads) values were determined for 25 maize *mTERF* genes in each of the zones and cells tested (Figure S7). In these genes, six genes showed higher expression levels with maximum RPKM >20 compared to *ZmTERF2*, *-3*, *-8*, *-9*, *-10*, *-13*, *-14*, *-15*, *-16*, *-21*, *-25* and *-26* with maximum RPKM<10. Of 25 maize *mTERF* genes, 16 were significantly differentially expressed along the leaf developmental gradient with CV >50% and *ZmTERF2*, *-10*, *-11*, *-12*, *-13*, *-15*, *-16*, *-21*, *-22*, *-26* and *-27* were down-regulated from the Base to Tip-1 zones. The result indicates that the *mTERF* genes were involved in leaf development and chloroplast biogenesis, especially required for early development of chloroplast. Ten *mTERF* genes (*ZmTERF2*, *-9*, *-10*, *-11*, *-13*, *-14*, *-15*, *-18*, *-25* and *-29*) were differentially expressed by >2-fold between bundle sheath and mesophyll cells. Notably, in the ten genes, only *ZmTERF29* was highly expressed with RPFM >20. These genes with different expression levels between bundle sheath and mesophyll cells are speculated to participate in coordination of chloroplast gene expression for C_4_ photosynthesis.

### Expression Profile of Maize *mTERF* Genes under Abiotic Stress

**Figure 7:**
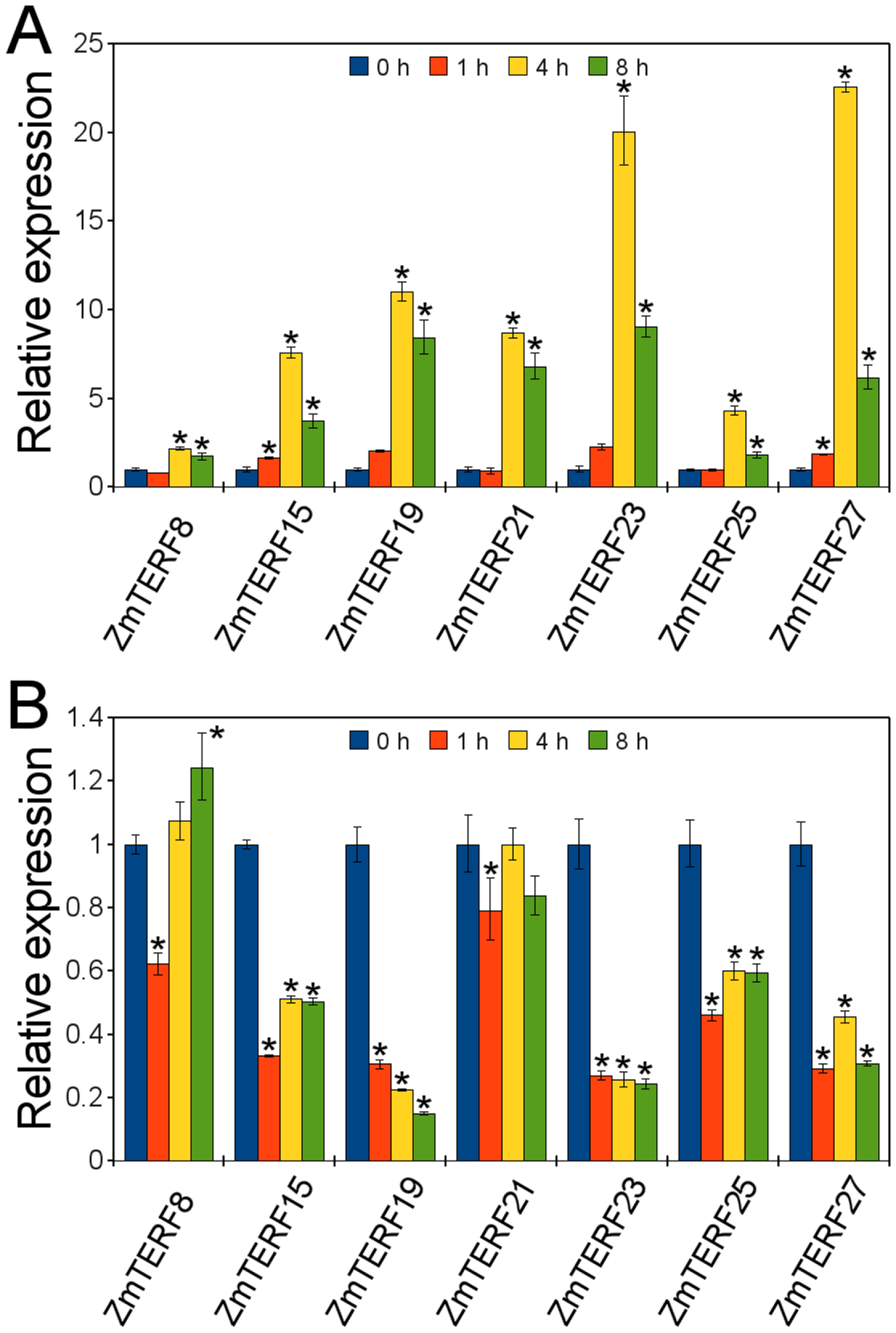
Differential expression of maize *mTERF* genes regulated by light/dark treatments. For the light treatment, total RNA was isolated from etiolated two-leaf seedling at 0, 1, 4 and 8 h after exposure to light (A). For the dark treatment, two-leaf seedlings cultured under 16 h light/8 h dark were used and placed in darkness after 24 h of continuous illumination (B). The expression levels were normalized against maize *Actin1* gene and the expression in the corresponding samples at 0 h was set as 1 using 2^–ΔΔCT^ method [75]. Error bars represent the standard error of the mean. Asterisk (*) on top of error bar indicate the significant difference (α = 0.05, n = 3) compared with control (0 h).

Previous reports on Arabidopsis *mTERF* genes have shown that loss of *mTERF* genes affect expression of stress-related genes [21], [24], phytohormone signal tranduction [17], and salt and osmotic stress tolerance [23], as well as organellar gene expression regulation. It was therefore envisioned that the maize mTERF genes may also be influenced by environmental signals. To examine the expression patterns of maize *mTERF* genes under various conditions including abiotic stress, several representative maize *mTERF* genes were selected for quantitative RT-PCR analysis under light/dark, salt and phytohormone treatments. Seven *mTERF* genes (*ZmTERF8*, *-15*, *-19*, *-21*, *-23*, *-25* and *-27*) were investigated under light and dark conditions – all these genes were up-regulated under light with peaks at 4 h after illumination and conversely, they were all down-regulated in darkness (Figure 7). In these genes, two chloroplast-targeting genes, *ZmTERF23* and *-27*, were largely up-regulated under light; and were also as well as *ZmTERF8*, heavily down-regulated in darkness by >2-fold (Figure 7). Plastids will develop from etioplasts to chloroplasts during de-etiolation of maize seedlings under illumination and photosynthetic apparatus will be developed. The result implied that *mTERF* genes are generally required for chloroplast development and photosynthesis in maize.

**Figure 8:**
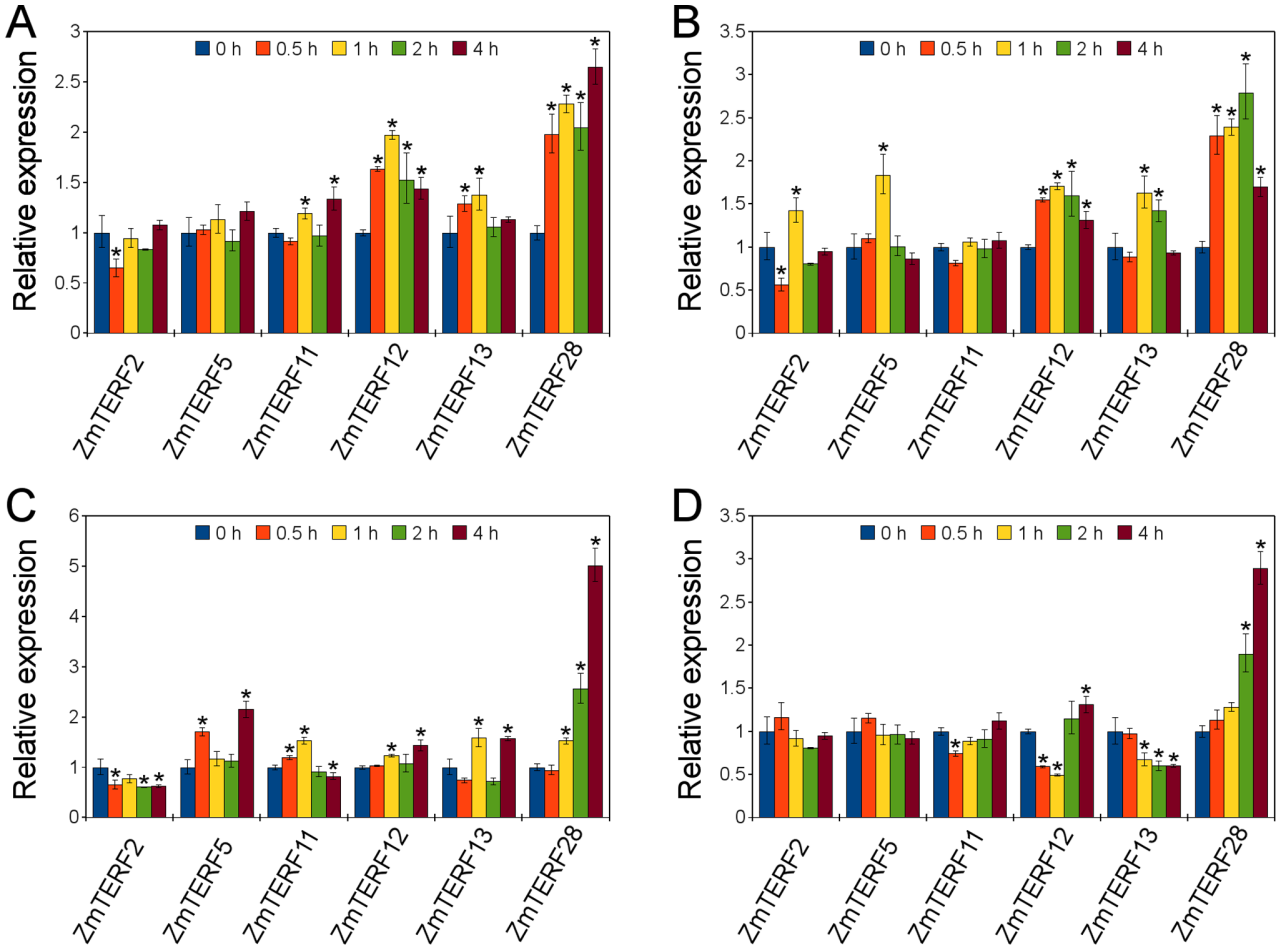
Quantitative RT-PCR analysis of maize *mTERF* gene expression under salts and phytohormones. Total RNA was extracted from two-leaf seedlings at 0.5, 1, 2 and 4 h after exposure to sodium chloride (A), aluminum chloride (B), NAA (C) and ABA (D), respectively. The expression levels were normalized against maize *Actin1* gene and the expression in the untreated control samples was set as 1 using 2^–ΔΔCT^ method [75]. Error bars indicate the standard error of the mean. Asterisk (*) on top of error bar represent the significant difference (α = 0.05, n = 3) compared with control (0 h).

Expression profiles of six maize mTERF genes at early stages (0–4 h) of response to salts (NaCl and AlCl_3_) and phytohormones (ABA and NAA) were analyzed (Figure 8). Of these six genes, *ZmTERF12* and *ZmTERF28* gene products were targeted to neither chloroplast nor mitochondria, while the remaining four genes (*ZmTERF2*, *-5*, *-11* and *-13*) all resided in mitochondria, with *ZmTERF5* and *ZmTERF11* of group IX. The expression changes of six *ZmTERFs* in response to salts and plant hormones were all less than 2 folds except *ZmTERF28* which was remarkably up-regulated with four stimuli (Figure 8). Expression levels of *ZmTERF2*, *-5* and *-11* were not affected by NaCl, AlCl_3_ and ABA treatments except those at certain time spots. For the *ZmTERFs* involved in stress response, *ZmTERF12* and *-13* showed similar expression patterns in response to NaCl and AlCl_3_, in which they were up-regulated up to 1 h followed by suppression in expression. For ABA treatment, *ZmTERF12* was down-regulated at first one hour after treatment and it was up-regulated thereafter, while the expression of *ZmTERF13* was suppressed after ABA treatment. For NAA treatment, the expression of all *ZmTERF* genes tested here were slightly changed at 2–3 time spots (Figure 8). These results suggested that maize *mTERF* genes, at least for these *ZmTERF* genes investigated here, had a limited role in response of maize to stress conditions.

## Discussion

To fully understand the role of *mTERF* genes in plants, it is therefore essential to characterize more *mTERF* genes in diverse plant species, particularly in crops. In this study, we report the systematic characterization of maize *mTERF* genes using bioinformatics and molecular biological approaches.

### Evolution of *ZmTERF* Genes

We carried out comprehensive searches for putative *ZmTERF* genes and found 29 *mTERF* genes in the sequenced maize genome, including a spliced gene model by genomic gap, and two extra *mTERF* genes identified in the NCBI Protein Database and cDNA library (Table S1). The two *ZmTERF* genes could not be mapped onto maize chromosomes. The number of *ZmTERF* genes was slightly less than for rice and Arabidopsis despite maize having a much larger genome. Therefore, the number of *ZmTERF* genes would be likely underestimated due to unsequenced genomic gaps or mis-annotation of gene models in MaizeGDB (http://www.maizegdb.org/) [55]. However, flowering plants have more *mTERF* genes than lower plants and mammals [18]. Given the complicated regulation of organellar genome transcription in higher plants [2], [3], [56], [57] and the conserved roles of Arabidopsis and *C. reinhardtii mTERF* genes in control of mitochondrial gene expression [5], expansion of the *mTERF* gene family in higher plants could be driven by complexity of organellar genomes.

Arabidopsis *mTERF* genes have been classified into eight groups based on phylogenetic relationships [17]. The phylogenetic tree including the *mTERF* proteins from maize, rice and Arabidopsis had similar topological structure to the tree constructed with *mTERF* genes of lower plants (Figure 1) [17]. Based on phylogenetic relationships, *ZmTERF* genes were divided into nine groups. All information about gene structure, motif distribution, subcellular location and protein size of *ZmTERF* genes support the conserved *ZmTERF* genes in the same groups shown in Figure 1. Group IX members shared group-specific motifs 2, 6, 7, 8 and 14, which confer novel functional characters compared with other groups. Only group IX was species-specific while the other groups were all composed of orthologs in various organisms used here, even including lower plants [17]. We conclude that group IX as well as VIII is newly generated before the divergence of angiosperms and have adopted a different evolutionary route from the other groups in plants; however, the other groups mainly containing chloroplast-targeting *mTERF* genes represent the ancestral family composition as supposed [17]. Sequence and evolutionary conservation leads to functional identification of Arabidopsis *mTERF* genes belonging to the ancestral groups (except for groups VIII and IX), since they are required for organellar gene expression as described for *hsmTERF*s.

It is largely accepted that the maize genome has undergone several rounds of genome duplication, including the latest whole genome duplication (WGD) about 5–12 million years ago (mya) after divergence from sorghum [55]. However, the WGD would not increase the amount of *ZmTERF* genes compared with sorghum, in which 38 members have been identified [18], suggesting that extension of the maize mTERF family cannot be explained by WGD alone. It is reported that nine Arabidopsis *mTERF* genes involved in tandem duplication were localized on chromosome 1 and formed a single cluster for these paralogs [18], whereas in the maize genome there were two tandemly duplicated *mTERF* gene clusters in chromosomes 1 and 5. The two clusters only contained five *ZmTERF* genes, representing 17% of *ZmTERF* genes, and are comparative with the four homologs generated by segmental duplication (Figure 4). Both segmental and tandem duplication are important for *mTERF* gene family expansion in maize, unlike for rice and Arabidopsis where tandem duplication played a predominant role in gene duplication [17], [18]. Duplicated genes may have three different evolutionary fates nonfunctionalization, subfunctionalization or neofunctionalization [58]. Sequence homology and gene expression analysis revealed that *ZmTERF4*, *-7* and *-20* were pseudogenes encoding truncated *mTERF* peptides and showed no expression in microarray data (Figure 6) and RT-PCR assay (Figure S2). This result showed that the three genes underwent nonfunctionalization by degenerative mutations during maize evolution. Two pairs of duplicates (*ZmTERF1* and *-29*, and *ZmTERF18* and *-19*) were stably preserved since they differ in their functions – such as differential spatial and temporal gene expression (Figure 6). The duplicated genes showed divergent expression patterns and have undergone neofunctionalization.

### Expression Divergence of *ZmTERF* Genes in Different Tissues or Organs

To establish organ and tissue-specific expression patterns of *ZmTERF* genes, NCBI ESTdb, available microarray and RNA-seq data were used. As shown in Figures 6, S5 and Table S7, most *ZmTERF* genes were actively transcribed as for Arabidopsis *mTERF* genes [18], and nine *ZmTERFs* were ubiquitously expressed in all organs and most tissues (Figure 6). In general, the temporal and tissue-specific expression of plastid-targeting *ZmTERF* genes was correlated with chloroplast development and photosynthesis proceeding, e.g. for *ZmTERF6*,*-9*, *-23*, *-24*, *-27* and *-29*. Expression divergence of *ZmTERF*s along the leaf developmental gradient and between mature bundle sheath and mesophyll cells were explored using RNA-seq data. The result reveals that quite a number of *ZmTERF*s showed differential expression levels among the four zones representing differentially developed chloroplasts, derived from developmental leaf, and ten *ZmTERF*s were differentially expressed between mature bundle sheath and mesophyll cells. Together, maize *mTERF* genes may have important roles in controlling organellar gene expression for chloroplast development and C_4_ photosynthesis.

Arabidopsis *BSM/RUG2* is essential for chloroplast biogenesis and development [17]. T-DNA insertion in SOLDAT10 causes lethal, whereas point mutation of SOLDAT10 shows lighter green cotyledons [21]. *SOLDAT10* can abrogate ^1^O_2_-mediated cell death of *flu* seedlings under illumination by perturbing chloroplast homeostasis and subsequently affecting the chloroplast-to-nucleus retrograde signaling pathway. The retrograde signals that adjust acclimation to light intensity are proposed to contribute to the signaling pathways that control photoperiodic acclimation of leaves [21]. Expression alteration of several *ZmTERF* genes in response to light and dark was investigated by qRT-PCR. Largely induced expression of chloroplast-targeting *ZmTERF23* and *-27* in response to light is understandable because these genes were probably requisite for transformation of plastid from etioplast to chloroplast during de-etiolation of maize seedling under illumination. The result is consistent with the presence of plentiful LREs in the corresponding promoters (Figure 5). Nevertheless, mitochondrial *ZmTERF* genes also show up-regulated expression in response to light. These genes might be required to optimize photosynthetic efficiency via coordinating the expression of mitochondrial genes and regulating the composition of the mitochondrial respiratory chain in the light, like *MOC1* in *C. reinhardtii* [19]. This hypothesis is also consistent with the down-regulated expression of these genes under dark treatment (Figure 7).

Selected *ZmTERF* genes show differential expression patterns under salt, ABA and NAA treatments. In Arabidopsis, most *mTERF* genes were heavily down-regulated in response to ABA, salt or mannitol [23]. In maize, however, *ZmTERF* genes showed different expression profiles to Arabidopsis *mTERF* genes, although several *ZmTERF* genes were down-regulated at certain periods after ABA treatment. This difference between Arabidopsis and maize *mTERF* genes might be due to the short-term response to ABA monitored in our study. Conversely, a few *ZmTERF* genes were up-regulated under salt treatments (AlCl_3_ and NaCl) to a substantial extent (Figure 8). The result may reflect the differences in response of monocots and dicots to salts. All of *ZmTERF* genes checked here were up or down-regulated with NAA at more than one time spot (Figure 8). This expression alteration is not consistent with the fact there were no known GREs in the promoters of these *ZmTERF* genes, and thus other unidentified elements in the promoter regions could account for the NAA-induced expression.

### Molecular Function of Plant *mTERF* Genes

In human, the biological roles of *mTERF* genes in controlling mitochondrial replication, transcription and translation have been well documented [10], [14]–[16]. Detailed molecular characterizations have shown that hsmTERF1 binds to mitochondrial DNA as a monomer [11], [37] and hsmTERF4 binds to mitochondrial rRNA within a protein complex containing NSUN4 [15], [16]. In *C. reinhardtii*, MOC1 protein can bind to the mitochondrial DNA specifically as does hsmTERF1 [19]. The conserved transcriptional factor-like function shared by *mTERF* genes in *C. reinhardtii* and human is probably responsible for the similar mechanism for mitochondrial genome transcription by which the mitochondrial genomes of human and *C. reinhardtii* are transcribed bidirectionally to produce two long primary transcripts, which are further processed to yield the mature mRNAs [19]. Increased *mTERF* members in higher plants may account for complicated regulation of mitochondrial genome as mtDNA transcription regulators besides chloroplast genome. Arabidopsis *mTERF* genes are required for normal expression of organellar genes [17], [21]–[24]. Moreover, plant mTERF proteins share conserved mTERF motif and similar 3D structure with human mTERF proteins (Figure 2) [17]. Arabidopsis mTERF protein, BSM/RUG2, has been shown to nonspecifically bind the chloroplast DNA segments [17]. It is envisaged that *mTERF* genes of higher plants have similar roles to *hsmTERF*s and *MOC1*. However, we cannot exclude the possibility that mTERF proteins of higher plants require auxiliary factors for specific DNA-binding because of existence of mTERF complex in chloroplast nucleoid [17], or these *mTERF* proteins have evolved the specialized property for RNA-binding, as has hsmTERF4 protein [15], [16], since Arabidopsis *mTERF* genes show co-expression with pentatricopeptide repeat (PPR) genes and might regulate mitochondrial RNA metabolism [18].

To date, only four *mTERF* genes of flowering plants have been functionally identified by a reverse genetic approach and all were Arabidopsis *mTERF* genes. Furthermore, three of the four genes are targeted to chloroplast [17], [21], [23] while only one (*SHOT1*) is targeted to mitochondria [24]. No known mutations have been reported so far for Arabidopsis groups VIII and IX genes (Figure 1). It is possible that these genes are functionally redundant or their mutations are lethal or conditional. Further biochemical evidence for the function of plant *mTERF* genes is still required to address these hypotheses. The biological functions of maize *mTERF* genes will be interpreted through mutant analysis, which is now accessible in the UniformMu Resource (http://www.maizegdb.org/documentation/uniformmu/) and the Mu Insertion Database (http://teosinte.uoregon.edu/mu-illumina/). Recently, several chloroplast-targeting *mTERF* mutants showing abnormal development of chloroplasts were observed and reported in the Photosynthetic Mutant Library (PML) (http://pml.uoregon.edu/pml_table.php) in which *ZmTERF27* cloned by Hammani and Brakan very recently is orthologous to Arabidopsis *BSM/RUG2* and plays an important role in group II intron splicing in maize chloroplasts [59]. In Arabidopsis, absence of *BSM/RUG2* causes abortion of embryo development, while in maize, mutants of *ZmTERF27* (*Zm-mterf4-1* and *Zm-mterf4-3* in PML) only causes disturbed leaf development of seedlings after seed germination, showing that in maize the effect of *ZmTERF27* mutation on embryo development can be alleviated [59]. This result may be due to variation of malonyl-coA synthase in maize and Arabidopsis [17].

## Conclusion

This is the first study of genome-scale analysis of *mTERF* genes in maize. We identifed 31 putative *ZmTERFs*, sorted them into nine groups based on their phylogenetic relationship, mined 15 conserved motifs, found contributions of gene duplications to the expansion of *mTERF* gene family in maize, and explored spatio-temporal and abiotic stress-responsive expression patterns of *ZmTERFs*. The results presented in this study provide basic information on maize *mTERF* genes and form the foundation for future functional studies of these proteins. Future functional analyses of maize *mTERF* genes identified in this study would eventually elucidate the biological mechanisms controlling organellar gene expression mediated by *ZmTERF* genes.

## Materials and Methods

### Identification of *mTERF* Genes in Maize

To obtain all putative *mTERF* family genes in maize, three approaches were used to mine maize expression and annotation databases. First, the keywords ‘maize’ and ‘mTERF’ were used to search the NCBI Protein database. Second, the reported sequences of the *mTERF1*-*mTERF4* genes in human [11], [12], [14], [15], *C. reinhardtti MOC1* [19] and Arabidopsis *mTERF*s [22] were collected and used as query sequences to search the maize filtered-gene set (ZmB73_5b_FGS_translations.fasta downloaded from www.maizesequence.org) in local BLASTP program [60] with E-value<1e-10. Additional local TBLASTN [60] was performed to query the Maize Full Length cDNA database (downloaded from http://www.maizecdna.org/) [61] and Unigene database of NCBI (www.ncbi.nlm.nih.gov) with the above known mTERF proteins. Third, conserved mTERF domains were used to query the maize filtered-gene set with mTERF HMM file (PF02536) from the Pfam database [62] using the HMMER 3.0 package [25], and the mTERF proteins were collected in terms of default inclusion threshold. Unique maize mTERF proteins were collected by manually eliminating redundant repeats. The maize mTERF proteins were confirmed within SMART [26]. Information about chromosomal localization, coding sequence length and aa length was obtained for each gene from MaizeGDB (http://maizegdb.org/). The molecular weight and theoretical isoelectric point (pI) value of the maize mTERFs were investigated within Expasy online tools (http://expasy.org/tools/).

### Gene Structure and Alternative Splicing

DNA and transcript sequences of maize *mTERF* genes were obtained from MaizeSequence (www.maizesequence.org) and MaizeGDB (http://maizegdb.org/) databases. There was more than one alternatively spliced transcript for most maize *mTERF*s annotated in the MaizeGDB database. Conserved gene structures of maize *mTERF*s were identified by comparing with their homologs in other higher plants in the CoGe program [63] and Phytozome v8.0 (http://www.phytozome.net). These conserved gene structures were drawn and displayed by Gene Structure Display Server (GSDS) [64].

To confirm the gene models of maize *mTERF*s, PCR amplification of maize *mTERF*s was performed with transcript specific primers (Table S2), and the DNA and cDNA derived from total RNA of B73 seedlings as a PCR template. PCR products were purified and sequenced in an ABI3730 sequencer (Sunny Bio., Shanghai). By comparing cDNA with DNA sequences, putative gene models were validated in the B73 inbred line.

### Motif and Conserved Sequence Analysis

Pfam [62] and SMART [26] database searching was carried out to identify known conserved motifs in maize mTERF proteins, and the Multiple Em for Motif Elicitation (MEME) program v4.9.0 [34] was used to predict the potential motifs in the putative *mTERF* family gene sequences with the following parameters: zero or one per sequence for distribution of a single motif, 6≤ optimum width of each motif ≤50, and maximum number of motifs to find = 15. mTERF motifs in maize *mTERF* proteins were collected and clustered using the ClustalW 2.0 program [33], and graphical representation of aa residues was made using WebLogo [65].

### Subcellular Location and Chromosomal Location of Maize *mTERF*s

TargetP [28] and Predotar [29] were used to predict subcellular location of maize mTERF proteins. Additionally, experimental proteomic data in the Plant Proteome DataBase (PPDB) for *Arabidopsis thaliana* and maize [30] were queried for validating the prediction results on the basis of appearance of mTERF proteins in specific organelle proteomes. Maize *mTERF* genes were mapped onto the corresponding maize chromosomes by identifying their chromosomal positions given in the MaizeGDB (http://maizegdb.org). Using MapChart software [66], the distribution of maize *mTERF* genes on chromosomes was drawn and modified manually with annotation information in MaizeGDB (http://maizegdb.org).

### Synteny Analysis and Gene Duplication

To compare the genomic context of maize *mTERF* genes with that in other grass species, information on their patterns of microsynteny was retrieved from the database Phytozome v8.0 (www.phytozome.net). Orthologs of maize *mTERF* genes in other grasses were investigated in the CoGe database [63]. Duplication analysis of maize *mTERF* genes was carried out in the Synteny Mapping and Analysis Program (SyMAP) v4.0 [44] and WGMapping program in the PLAZA v2.5 platform [45]. Two *mTERF*s placed on the synteny blocks in maize genome were designated as segmental duplicated paralogs, and two *mTERF*s separated by five or fewer gene loci were regarded as tandem duplicated paralogs as described [67].

### Sequence Alignment and Phylogenetic Tree Construction

A total of 98 aa sequences (Table S3) of the *mTERF* genes were aligned with the program MUSCLE [68]. The resulting alignment was manually optimized by removing unaligned residues in CINEMA 5 (http://aig.cs.man.ac.uk/research/utopia/cinema/cinema.php). A Maximum Likelihood (ML) phylogenetic tree was constructed using RAxML v7.2.8 [27] with the JTT aa substitution model. Bootstrap testing was performed with 500 re-sampling to search for the best tree. The ML phylogenetic tree was depicted by FigTree v1.4.0 (http://tree.bio.ed.ac.uk/software/-figtree/).

### Secondary Structure and 3D Structure Analysis

To interpret the responsibility of conserved higher structure for roles of *mTERF* genes in plant organellar gene expression regulation, the secondary structures of mTERF proteins were analyzed using MINNOU [69], and their 3D structures were remodeled using the I-TASSER program [42] on the basis of crystal structure of previously resolved human mTERFs [16], [36]–[37], [70]. Superimposing 3D structures of maize mTERFs with that of human mTERFs was performed in the TM-align program [43]. PyMOL v1.6.0.0 [71] was used to display and analyze the tertiary structure of maize mTERFs.

### *Cis*-Regulatory Elements in Promoter

Promoter sequences (2-kb upstream of the translation start codon) for maize mTERF genes were obtained from the MaizeSequence database (http://www.maizesequence.org/-index.html) and subjected to prediction of *cis*-acting regulatory DNA elements using PlantPAN [49] with the transcription factors selected from rice, maize and Arabidopsis. The regulatory DNA elements were displayed in Argo Genome Browser v1.0.26 (http://www.broadinstitute.org/annotation/argo/).

### Gene Expression Profile Analysis

Spatio-temporal expression regulation of maize *mTERF*s was investigated using the microarray data (ZM37) for a genome-wide gene expression altas of the entire life cycle of B73 maize [50] from PLEXdb [72]. Robust Multi-array Average (RMA) normalized and log_2_-transformed expression data of maize *mTERF*s was retrieved from ZM37 and median expression values were loaded into Cluster 3.0 [51] for hierarchical clustering analysis. Clustered expression data of maize mTERFs were depicted using Java TreeView v1.1.5 [73]. eFP browser [53] was used to analyze the expression regulation of maize *mTERF*s during seedling leaf development as well as in bundle sheath and mesophyll cells.

### Plant Stress Treatment

To verify the expression regulation of *mTERF* genes in maize under hormone and salt [sodium chloride (NaCl, 200 mM) and aluminum chloride (AlCl_3_, 200 mM)] treatments, the two-leaf B73 seedlings were cultured in water under the following chemical treatments, ABA (100 μM) and 1-Naphthaleneacetic acid (NAA, a synthetic plant hormone in the auxin family) (100 μM) (Sigma-Aldrich, Shanghai), respectively. Samples were collected at 0.5, 1, 2 and 4 h after the above treatment, with three biological replicates per sample. To investigate effects of light on expression of maize *mTERF*s, etiolated B73 seedlings cultured in darkness were placed under continuous light, while normal-cultured B73 seedlings cultured under 16 h light/8 h dark were placed in darkness after 24 h of continuous light. Samples were collected at 1, 2, 4 and 8 h after light or dark treatment, with three biological replicates per sample. Total RNAs of collected samples were isolated with Trizol reagent (Invitrogen, USA) according to the manufacturer’s instructions and quantified by NanoDrop 2000 spectrophotometer (ThermoFisher, USA) before cDNA synthesis.

### Quantitative Real-Time PCR Analysis

For real-time PCR analysis, first-strand cDNA was synthesized from RNase-free DNase I (Fermentas, USA)-treated total RNA using Superscript II reverse transcriptase (Invitrogen, USA) according to the manufacturer’s instructions. Real-time PCR was performed in an optical 96-well plate with a BioRad CFX96 Real-time PCR System (Bio-Rad Laboratories, Inc.) with the gene-specific primers (Table S9). Each reaction contained 12.5 μl of 2×SYBR Green Master Mix Reagent (Applied Biosystems, USA), 5 μl of diluted cDNA sample and 500 nM of each primer (Table S2) in a final volume of 25 μl. The thermal cycle used was as follows: 95°C for 5 min; 45 cycles of 95°C for 15 s and 60°C for 15 s. The maize *Actin1* gene (GenBank accession number, J01238) was used as the internal control [74]. The relative expression levels were determined using 2^–ΔΔCT^ method as described previously [75]. The SAS v9.3 (SAS Institute Inc., USA) was used for the statistical analysis and the Dunnett’s t test was used to compare the significant difference of all stress treatments against their controls.

## Supporting Information

Figure S1 **Correction of** ***ZmTERF23*** **gene model.** (A) Multiple sequence alignment for *GRMZM2G426154*, *GRMZM2G175930* and *BT084020* was performed in Clustal Omega (http://www.ebi.ac.uk/Tools/msa/clustalo/). Identical nucleotides are denoted by asterisks under the alignment. (B) BAC contigs were rearranged to produce correct *ZmTERF23*.
(TIFF)

Figure S2 **Exon–intron organization of maize mTERF genes.** The gene structures of maize mTERF genes were generated by comparing the transcript sequences with corresponding DNA sequences in GSDS [64]. Classification and subcellular localization information of maize mTERF genes shown in this picture were derived from Figure 1 and Table 1, respectively.
(TIFF)

Figure S3 **Schematic structure of maize mTERF proteins.** mTERF motifs identified in SMART [26] are boxed in black. Light gray boxes denote mTERF motifs with lower reliability.
(TIFF)

Figure S4 **Validation of maize** ***mTERF*** **gene models by RT-PCR and DNA sequencing.** DNA and cDNA derive from total RNA isolated from B73 seedling leaf were used as templates to amplify the maize *mTERF* genes with transcript-specific primers listed in. Each of maize mTERF genes was amplified and surveyed in 1% agarose gel followed by EB (ethidium bromide) staining.
(TIFF)

Figure S5 **Multiple sequence alignment of ZmTERF27 and its homologs in Arabidopsis, rice and human.** MUSCLE program [68] was used to align the amino acid sequences of ZmTERF27, BSM/RUG2, LOC_Os05g33500 and HsmTERF1. The secondary structure of ZmTERF27 protein displayed under the aligned sequences was predicted in MINNOU [69]. mTERF motifs (M1–M9) are shown as black bars under their conserved residues. Putative mTERF structure modules with two or three α-helices are represented by red bars. Conserved arginine residues are highlighted by red asterisks.
(TIFF)

Figure S6 **Expression patterns of maize mTERF genes in different tissues analyzed by realtime RT-PCR.** Total RNA was isolated from seedling leaf, root, unpollinated silk, mature seed and immature tassel of B73 maize. Three biological replicates per sample were conducted. The expression levels of each gene were normalized against *Actin1* gene using 2^–ΔCt^ methods [75]. Duncan’s multiple range test (MRT) was used to statistically analyze the expression in different tissues and significant differences were marked by little letter on the top of bars (α = 0.05, n = 3). Error bars indicate the standard error of the mean.
(TIFF)

Figure S7 **Expression levels of maize mTERF genes in B73 seedling leaf.** Transcript abundance of maize mTERF genes in different developmental gradients and cells of B73 seedling leaf were retrieved from eFP browser (http://bar.utoronto.ca/efp_maize/cgi-bin/efpWeb.cgi) [53]. Base, base of the leaf; Ligule+4, –1 cm from ligule; Ligule+9, +4 cm from ligule; Tip_–1, –1 cm from tip; Tip_BS, bundle sheath cells of Tip_–1; Tip_ME, mesophyll cells of Tip_–1. Expression levels (RPKM) of maize *mTERF* genes are represented on y-axis.
(TIFF)

Table S1 **List of maize** ***mTERF*** **genes identified from three databases.**
(XLS)

Table S2 **Characterization of maize** ***mTERF*** **genes and proteins in detail.**
(XLS)

Table S3 ***mTERF*** **genes of rice and** ***Arabidopsis thaliana*** **and other known** ***mTERF*** **genes used for construction of the phylogenetic tree in this study.**
(DOC)

Table S4 **Transcript-specific primers used for gene model validation of maize** ***mTERF*** **genes.**
(DOC)

Table S5 **Annotation of** ***cis*** **-regulatory elements involved in phytohormone and abiotic stress response of plants.**
(XLS)

Table S6 ***Cis*** **-regulatory elements found in the 2-kb upstream regions of maize mTERF genes.**
(XLS)

Table S7 **Digital expression analysis of maize** ***mTERF*** **genes.**
(DOC)

Table S8 **Spatio-temporal expression levels of maize mTERF genes.**
(XLS)

Table S9 **Gene-specific primers used for qRT-PCR analysis of maize** ***mTERF*** **genes.**
(DOC)

File S1 **FASTA format multiple sequence alignment of human and plant mTERF proteins.**
(FAS)
