# Supplementary figures and images for: Genome-Wide Identification, Evolution and Expression Analysis of mTERF Gene Family in Maize
Source: PLoS One. 2014 Apr 9;9(4):e94126. doi: 10.1371/journal.pone.0094126 (PMC3981765; doi:10.1371/journal.pone.0094126)

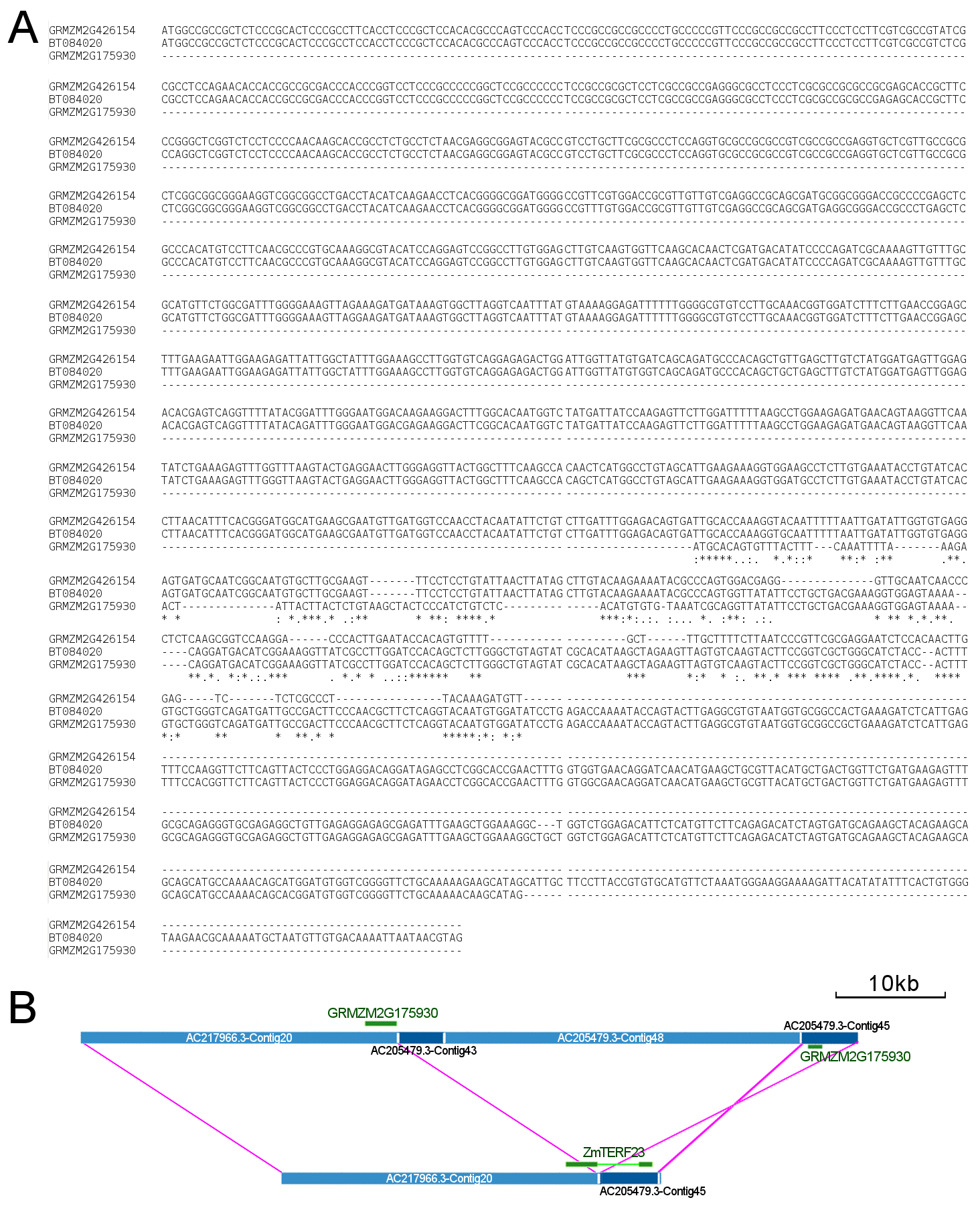

Supplement: Figure S1 — Correction of ZmTERF23 gene model. (A) Multiple sequence alignment for GRMZM2G426154, GRMZM2G175930 and BT084020 was performed in Clustal Omega (http://www.ebi.ac.uk/Tools/msa/clustalo/). Identical nucleotides are denoted by asterisks under the alignment. (B) BAC contigs were rearranged to produce correct ZmTERF23. (TIFF) [file pone.0094126.s001.tif]

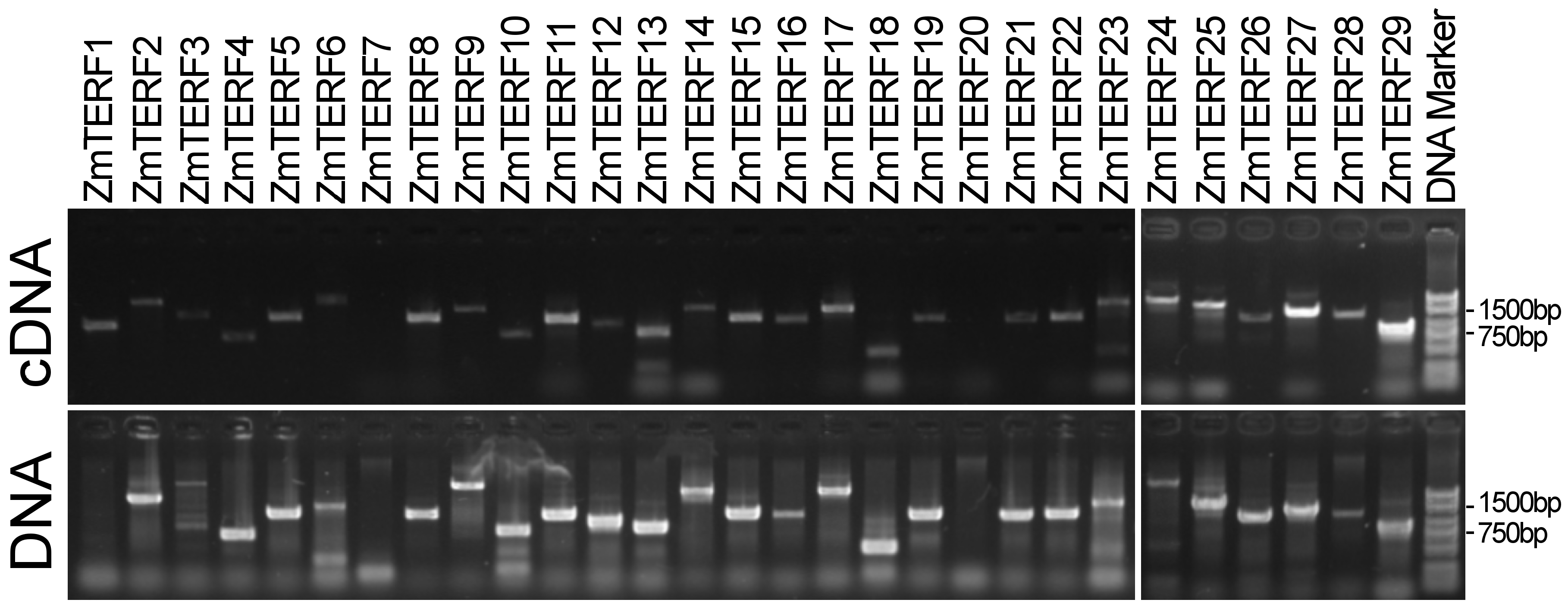

Supplement: Figure S2 — Exon–intron organization of maize mTERF genes. The gene structures of maize mTERF genes were generated by comparing the transcript sequences with corresponding DNA sequences in GSDS [64]. Classification and subcellular localization information of maize mTERF genes shown in this picture were derived from Figure 1 and Table 1, respectively. (TIFF) [file pone.0094126.s002.tif]

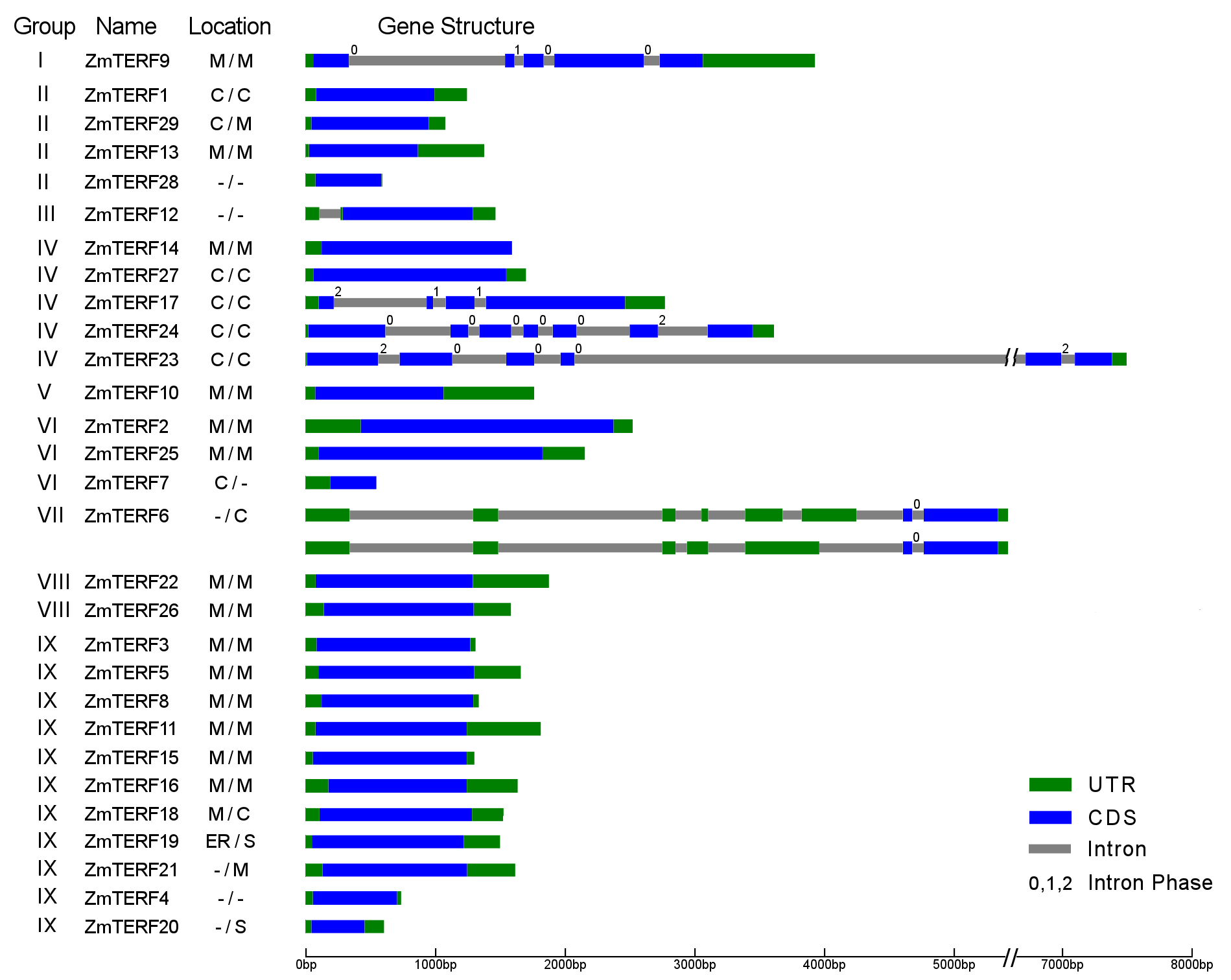

Supplement: Figure S3 — Schematic structure of maize mTERF proteins. mTERF motifs identified in SMART [26] are boxed in black. Light gray boxes denote mTERF motifs with lower reliability. (TIFF) [file pone.0094126.s003.tif]

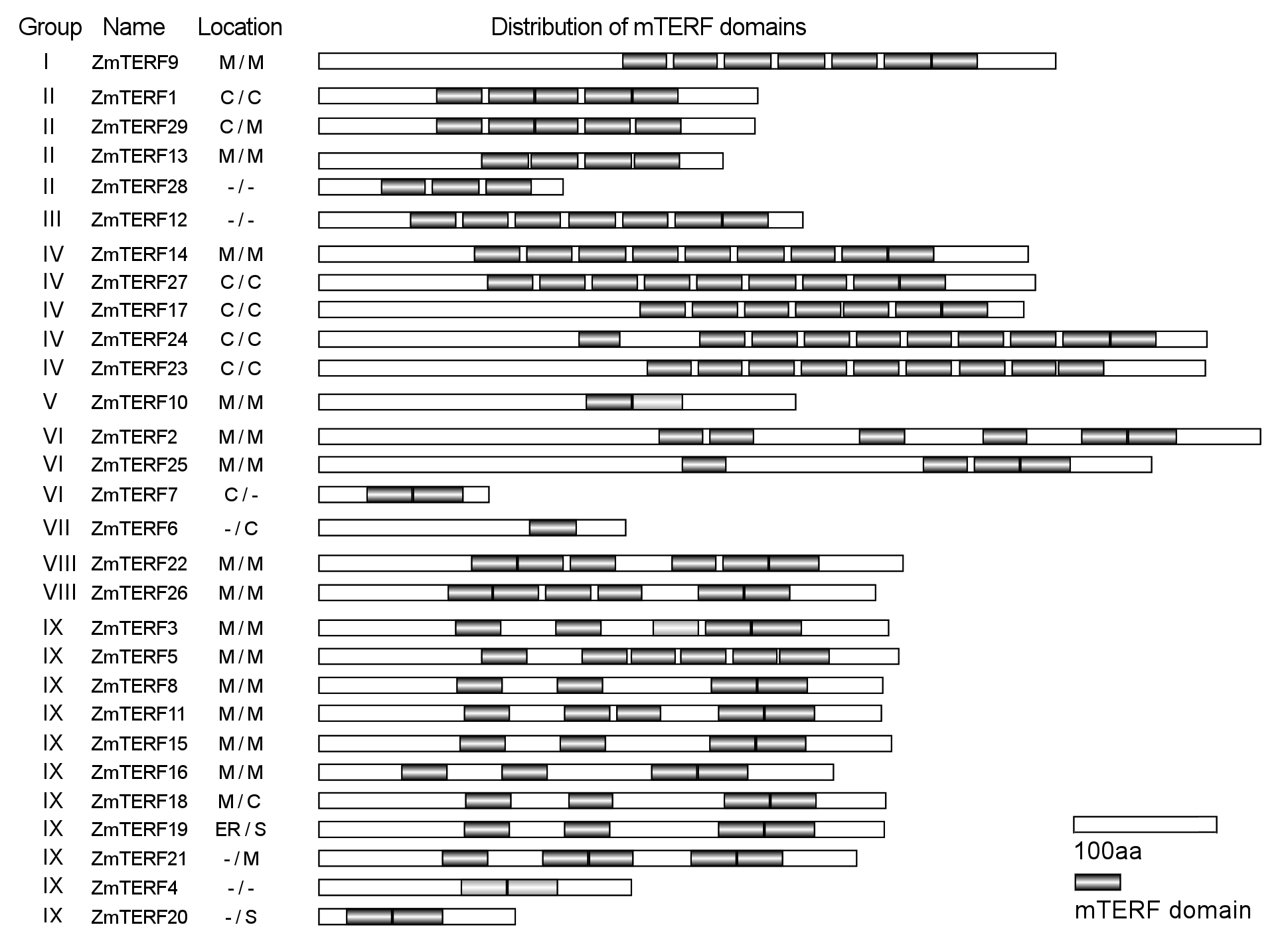

Supplement: Figure S4 — Validation of maize mTERF gene models by RT-PCR and DNA sequencing. DNA and cDNA derive from total RNA isolated from B73 seedling leaf were used as templates to amplify the maize mTERF genes with transcript-specific primers listed in. Each of maize mTERF genes was amplified and surveyed in 1% agarose gel followed by EB (ethidium bromide) staining. (TIFF) [file pone.0094126.s004.tif]

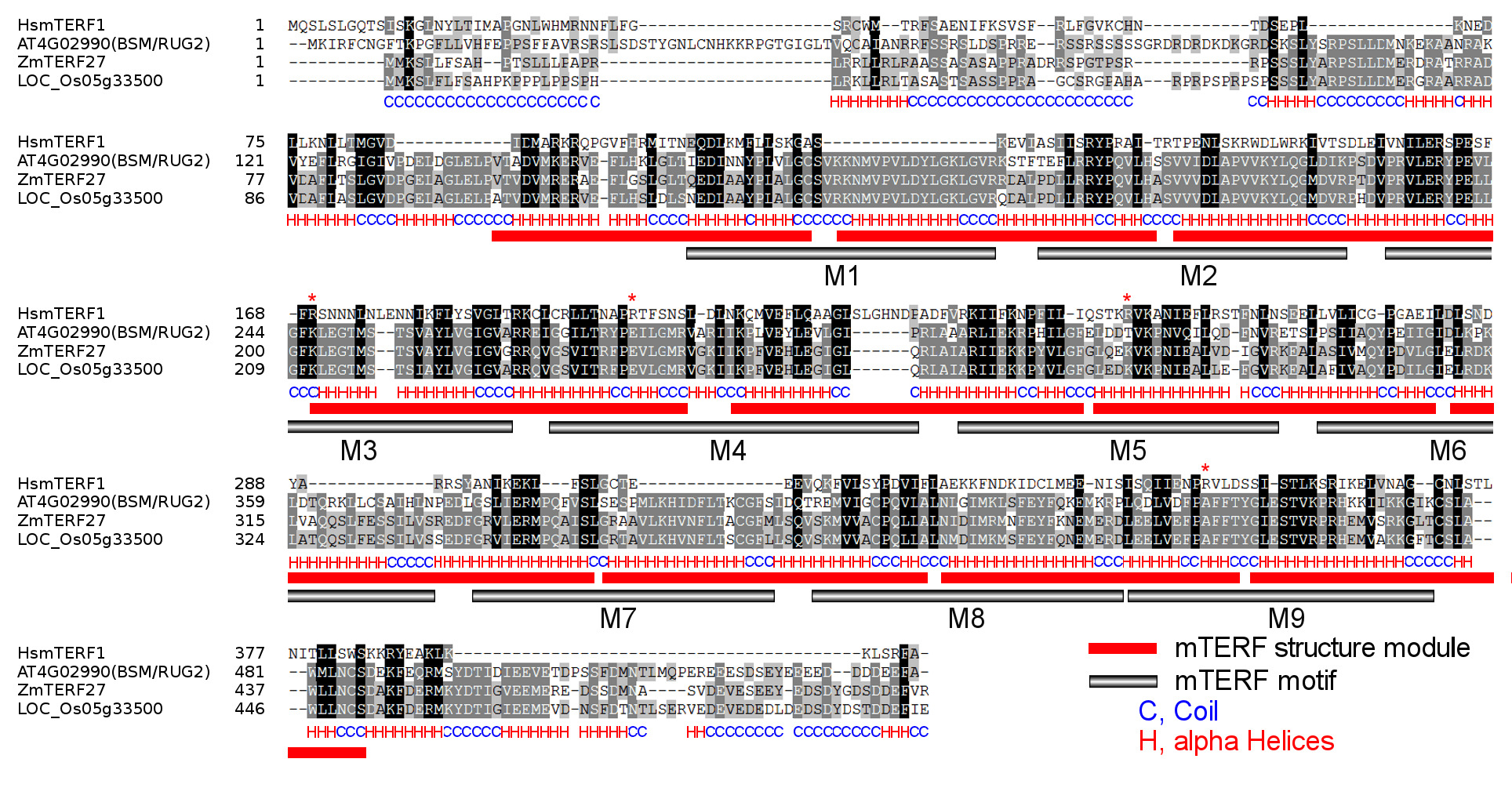

Supplement: Figure S5 — Multiple sequence alignment of ZmTERF27 and its homologs in Arabidopsis, rice and human. MUSCLE program [68] was used to align the amino acid sequences of ZmTERF27, BSM/RUG2, LOC_Os05g33500 and HsmTERF1. The secondary structure of ZmTERF27 protein displayed under the aligned sequences was predicted in MINNOU [69]. mTERF motifs (M1–M9) are shown as black bars under their conserved residues. Putative mTERF structure modules with two or three α-helices are represented by red bars. Conserved arginine residues are highlighted by red asterisks. (TIFF) [file pone.0094126.s005.tif]

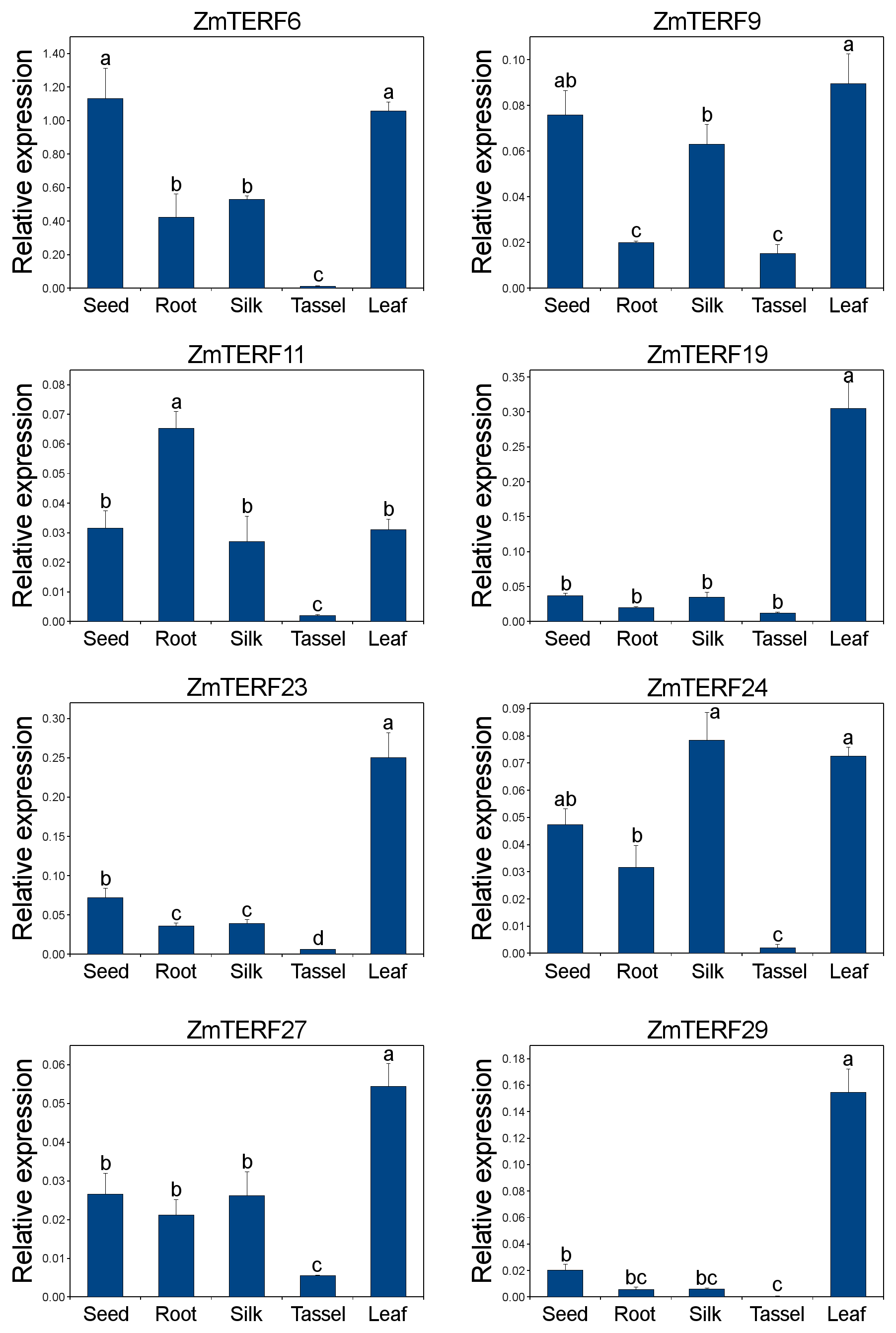

Supplement: Figure S6 — Expression patterns of maize mTERF genes in different tissues analyzed by realtime RT-PCR. Total RNA was isolated from seedling leaf, root, unpollinated silk, mature seed and immature tassel of B73 maize. Three biological replicates per sample were conducted. The expression levels of each gene were normalized against Actin1 gene using 2–ΔCt methods [75]. Duncan’s multiple range test (MRT) was used to statistically analyze the expression in different tissues and significant differences were marked by little letter on the top of bars (α = 0.05, n = 3). Error bars indicate the standard error of the mean. (TIFF) [file pone.0094126.s006.tif]

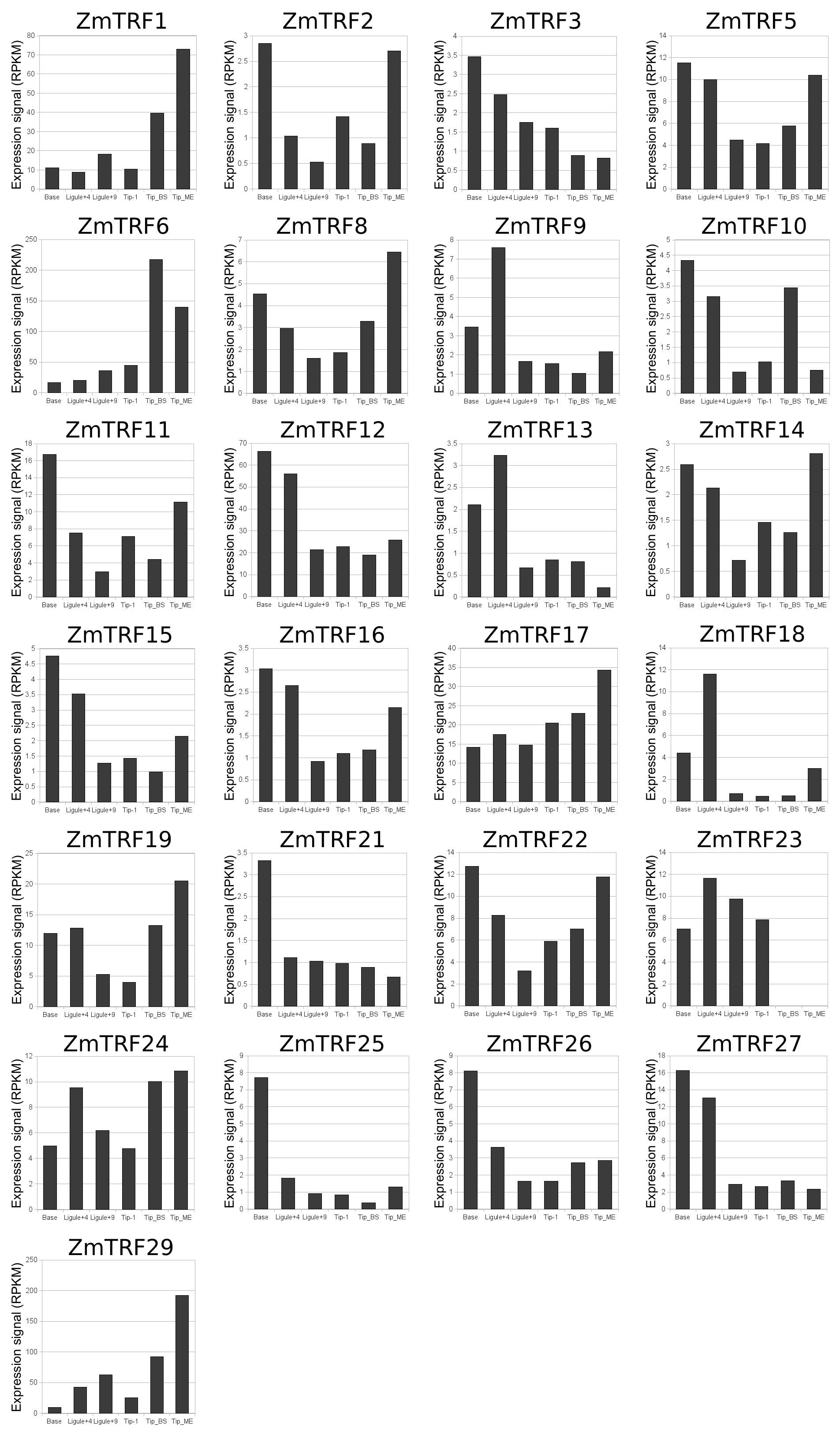

Supplement: Figure S7 — Expression levels of maize mTERF genes in B73 seedling leaf. Transcript abundance of maize mTERF genes in different developmental gradients and cells of B73 seedling leaf were retrieved from eFP browser (http://bar.utoronto.ca/efp_maize/cgi-bin/efpWeb.cgi) [53]. Base, base of the leaf; Ligule+4, –1 cm from ligule; Ligule+9, +4 cm from ligule; Tip_–1, –1 cm from tip; Tip_BS, bundle sheath cells of Tip_–1; Tip_ME, mesophyll cells of Tip_–1. Expression levels (RPKM) of maize mTERF genes are represented on y-axis. (TIFF) [file pone.0094126.s007.tif]
