# Supplementary material for: Genome-Wide Identification, Evolution and Expression Analysis of mTERF Gene Family in Maize
Source: PLoS One. 2014 Apr 9;9(4):e94126. doi: 10.1371/journal.pone.0094126 (PMC3981765; doi:10.1371/journal.pone.0094126)
Supplement: Table S3 — mTERF genes of rice and Arabidopsis thaliana and other known mTERF genes used for construction of the phylogenetic tree in this study. (DOC) [file pone.0094126.s010.doc]

**Table S3. mTERF genes of rice and *Arabidopsis thaliana* and other known *mTERF*** genes used for construction of phylogenetic tree in this study.

| Species | Gene Name | Genebank No.^a^ | Protein Length (aa) | ORF Length (bp) | Subcellular Location^b^ |
| --- | --- | --- | --- | --- | --- |
| *H. sapiens* | *HsmTERF1* | Q99551 | 399 | 1200 | M |
|  | *HsmTERF2* | Q49AM1 | 385 | 1158 | M |
|  | *HsmTERF3* | Q96E29 | 417 | 1254 | M |
|  | *HsmTERF4* | Q7Z6M4 | 381 | 1146 | M |
| *C. reinhardtti* | *Cre_MOC1* | AAM96690 | 373 | 1122 | M |
| *O. sativa* ssp. | *LOC_Os01g27690* | EEC70669 | 139 | 420 | C/C |
| japonica | *LOC_Os02g36780* | NP_001173034 | 282 | 849 | C/M |
|  | *LOC_Os02g39040* | - | 490 | 1473 | C/C |
|  | *LOC_Os02g51450* | BAD15633 | 399 | 1200 | M/ER |
|  | *LOC_Os02g51460* | NP_001048129 | 382 | 1149 | M/M |
|  | *LOC_Os02g54200* | EEE57928 | 574 | 1725 | M/M |
|  | *LOC_Os03g24590* | NP_001050155 | 301 | 906 | M/C |
|  | *LOC_Os03g57149* | NP_001051481 | 310 | 933 | C/C |
|  | *LOC_Os03g62240* | - | 214 | 645 | -/M |
|  | *LOC_Os04g54510* | NP_001054025 | 393 | 1182 | M/M |
|  | *LOC_Os05g33440* | NP_001055498 | 393 | 1182 | M/M |
|  | *LOC_Os05g33460* | NP_001055499 | 399 | 1200 | M/M |
|  | *LOC_Os05g33500* | NP_001055500 | 508 | 1527 | C/C |
|  | *LOC_Os05g34160* | NP_001055544 | 395 | 1188 | M/ER |
|  | *LOC_Os06g12040* | NP_001057188 | 392 | 1179 | M/M |
|  | *LOC_Os06g12050* | - | 324 | 975 | M/M |
|  | *LOC_Os06g12060* | BAD38193 | 875 | 2628 | M/M |
|  | *LOC_Os06g12070* | NP_001057191 | 393 | 1182 | M/M |
|  | *LOC_Os06g12080* | NP_001057192 | 378 | 1137 | M/M |
|  | *LOC_Os06g12100* | BAD37286 | 404 | 1215 | M/M |
|  | *LOC_Os06g12110* | NP_001057195 | 393 | 1182 | M/M |
|  | *LOC_Os07g04230* | NP_001058844 | 608 | 1827 | M/C |
|  | *LOC_Os07g22670* | NP_001059441 | 575 | 1728 | M/M |
|  | *LOC_Os07g24090* | NP_001059486 | 408 | 1227 | M/M |
|  | *LOC_Os07g39430* | NP_001060114 | 503 | 1512 | C/ER |
|  | *LOC_Os08g40430* | NP_001062235 | 333 | 1002 | -/- |
|  | *LOC_Os08g40630* | NP_001062250 | 636 | 1911 | M/M |
|  | *LOC_Os09g38720* | BAD45958 | 651 | 1956 | C/ER |
|  | *LOC_Os11g09990* | NP_001067457 | 406 | 1221 | M/M |
|  | *LOC_Os11g10000* | NP_001176421 | 458 | 1377 | M/M |
|  | *LOC_Os11g10040* | NP_001067460 | 392 | 1179 | M/M |
|  | *LOC_Os11g14130* | NP_001067605 | 417 | 1254 | C/M |
|  | *LOC_Os12g30610* | ABA98287 | 332 | 999 | M/M |
| *A. thaliana* | *AT1G21150* | NP_173539 | 390 | 1173 | M |
|  | *AT1G56380* | NP_176034 | 388 | 1167 | - |
|  | *AT1G61960* | NP_176387 | 457 | 1374 | M |
|  | *AT1G61970* | NP_176388 | 418 | 1257 | M |
|  | *AT1G61980* | NP_176389 | 418 | 1257 | M |
|  | *AT1G61990* | NP_176390 | 414 | 1245 | M |
|  | *AT1G62010* | NP_176392 | 415 | 1248 | M |
|  | *AT1G62085* | NP_974069 | 461 | 1386 | - |
|  | *AT1G62110* | NP_176402 | 462 | 1389 | M |
|  | *AT1G62120* | NP_176403 | 437 | 1314 | M |
|  | *AT1G62150* | NP_176406 | 463 | 1392 | M |
|  | *AT1G62490* | NP_176438 | 334 | 1005 | - |
|  | *AT1G74120* | NP_565080 | 445 | 1338 | M |
|  | *AT1G78930* | NP_178014 | 591 | 1776 | C |
|  | *AT1G79220* | NP_565202 | 399 | 1200 | M |
|  | *AT2G03050* | NP_178405 | 283 | 852 | C |
|  | *AT2G21710* | NP_179763 | 641 | 1926 | C |
|  | *AT2G34620* | NP_181009 | 303 | 912 | C |
|  | *AT2G36000* | NP_565830 | 333 | 1002 | C |
|  | *AT2G44020* | NP_566005 | 507 | 1524 | M |
|  | *AT3G18870* | NP_188517 | 274 | 825 | C |
|  | *AT3G46950* | NP_190279 | 450 | 1353 | M |
|  | *AT3G60400* | NP_191599 | 558 | 1677 | M |
|  | *AT4G02990* | NP_192208 | 541 | 1626 | C |
|  | *AT4G09620* | NP_192700 | 212 | 639 | C |
|  | *AT4G14605* | NP_567435 | 493 | 1482 | C |
|  | *AT4G19650* | NP_193700 | 575 | 1728 | - |
|  | *AT4G38160* | NP_195529 | 333 | 1002 | M |
|  | *AT5G06810* | NP_196299 | 1141 | 3426 | - |
|  | *AT5G07900* | NP_568185 | 405 | 1218 | M |
|  | *AT5G23930* | NP_197781 | 457 | 1374 | - |
|  | *AT5G45113* | NP_680395 | 414 | 1245 | C&N |
|  | *AT5G54180* | NP_200229 | 500 | 1503 | C |
|  | *AT5G55580* | NP_200369 | 496 | 1491 | C |
|  | *AT5G64950* | NP_201300 | 391 | 1176 | M |

^a^ Corresponding protein accession numbers in NCBI (http://www.ncbi.nlm.nih.gov/). There are two gene models for *MOC1*, enconding 251aa (EDP02860) and 373aa (AAM96690) proteins, respectively [19,20]. The AAM96690 protein was used in this study. The sequences of several rice mTERF proteins deposited in NCBI are different from those deduced in Phytozome (http://www.phytozome.net/). However, here we used the protein sequences annotation in Phytozome for phylogenetic analysis.

^b^ The subcellular localization information of mTERF proteins is from corresponding references for *H. sapiens*, *C. reinhardtti*, and *A. thaliana*, but not for *O. sativa* ssp. japonica which mTERFproteins were subject to TargetP [28] and (/) Predotar [29] tools for subcellular localization prediction. M, mitochondria; C, chloroplast; N, nuclear; S/ER, secretory protein; -, other places.
