# Supplementary material for: Genome-Wide Identification, Evolution and Expression Analysis of mTERF Gene Family in Maize
Source: PLoS One. 2014 Apr 9;9(4):e94126. doi: 10.1371/journal.pone.0094126 (PMC3981765; doi:10.1371/journal.pone.0094126)
Supplement: Table S4 — Transcript-specific primers used for gene model validation of maize mTERF genes. (DOC) [file pone.0094126.s011.doc]

**Table S4**. Transcript-specific primers used for gene model validation of maize mTERF genes.

| Gene Model^a^ | Primer Name | Primer Sequence (5'->3') | Length of PCR Amplicon (bp)^b^ | |
| --- | --- | --- | --- | --- |
|  |  |  | DNA | cDNA |
| GRMZM2G168665 | ZmTERF1-F | TCGACCAGATCCATCTCACA | 1041 | 1041 |
| (ZmTERF1) | ZmTERF1-R | ACACCACCAAGAGGCAAGAG |  |  |
| GRMZM2G061542 | ZmTERF2-F | CTAGCTTGGTTTCCCCATGA | 1998 | 1998 |
| (ZmTERF2) | ZmTERF2-R | CCCACTGCCTTTTCCATAAA |  |  |
| GRMZM2G034217 | ZmTERF3-F | GATGCTTCGGTTGTGGAGTT | 3267 | 1351 |
| (ZmTERF3) | ZmTERF3-R | ACCCAATGCTCTTTTTGGTG |  |  |
| GRMZM2G054517 | ZmTERF4-F | CACCCACTTAACCAACAAGG | 739 | 739 |
| (ZmTERF4) | ZmTERF4-R | CTGCCAAGTGCCTACAACAT |  |  |
| GRMZM2G159766 | ZmTERF5-F | CGACGACCTAATCCCCTTC | 1310 | 1310 |
| (ZmTERF5) | ZmTERF5-R | GCATCCACCTAAAACACAAAA |  |  |
| GRMZM2G170137 | ZmTERF6-F | ACAGAGAGTGGAAACCGGAA | 5195 | 1921/1948^d^ |
| (ZmTERF6) | ZmTERF6-R | ACAGAAAAATGGCCAGCAAG |  |  |
| GRMZM2G087679 | ZmTERF7-F | GTTGGGTATGACCACCAAGG | 382 | 382 |
| (ZmTERF7) | ZmTERF7-R | TAATTATGGTCCCCGACAGG |  |  |
| GRMZM2G060114 | ZmTERF8-F | TCTTCGTTTCCTCCTCATCG | 1288 | 1288 |
| (ZmTERF8) | ZmTERF8-R | CCAGCTCACTGCTAAGTGTCA |  |  |
| GRMZM2G130773 | ZmTERF9-F | CCCCAAAAAGAAATATCCAAGG | 3101 | 1622 |
| (ZmTERF9) | ZmTERF9-R | CGTGGGCTCAAGAGAAACA |  |  |
| GRMZM2G177019 | ZmTERF10-F | ATTCTGATCATGGCCACCAC | 834 | 834 |
| (ZmTERF10) | ZmTERF10-R | CTTCATTTCAACGGGGTCTC |  |  |
| GRMZM2G023257 | ZmTERF11-F | GTGCTGCTGGAGGTTTGTC | 1273 | 1273 |
| (ZmTERF11) | ZmTERF11-R | GCTGCCCATATAGCCAGTTT |  |  |
| GRMZM2G113181 | ZmTERF12-F | CGCTAGCTATCCCCTCATCG |  |  |
| (ZmTERF12) | ZmTERF12-R | TACCTAAACTGCTGCGACCA | 1481 | 1115 |
| GRMZM2G312806 | ZmTERF13-F | AAAGCCCCAAGCCAAATC | 890 | 890 |
| (ZmTERF13) | ZmTERF13-R | AAGGCTTAGGGTGCATCTGA |  |  |
| GRMZM2G000610 | ZmTERF14-F | CTTCGTCACGGCCCAAAG | 2540 | 1737 |
| (ZmTERF14) | ZmTERF14-R | AGGGCAGCACTCAAACTACT |  |  |
| GRMZM2G119921 | ZmTERF15-F | CGGAACACTGGTAACCCTCA | 1284 | 1284 |
| (ZmTERF15) | ZmTERF15-R | AAAAACAAGGCTTGCAGCAG |  |  |
| GRMZM2G395850 | ZmTERF16-F | CTTCTTTTCGCCACCAAATG | 1270 | 1270/1168^d^ |
| (ZmTERF16) | ZmTERF16-R | AGAAGGGAGGCAGCAAGATA |  |  |
| GRMZM2G024550 | ZmTERF17-F | TTCTCGATAGCCTCTCTCTCC |  |  |
| (ZmTERF17) | ZmTERF17-R | CGTGCTCTGAAAACTGATGG | 2533 | 1631 |
| GRMZM2G017355 | ZmTERF18-F | CACACCTGTCAGCACAACCT | 1304 | 1304 |
| (ZmTERF18) | ZmTERF18-R | ACAGGGATCATCAGCTCTCC |  |  |
| GRMZM2G017429 | ZmTERF19-F | GCCATGCCTCTCATCTTCTT | 1228 | 1228 |
| (ZmTERF19) | ZmTERF19-R | AAATGCACAATGGAGGGAAC |  |  |
| GRMZM2G158854 | ZmTERF20-F | GACAGGTTGTGTTCGTCGTG | 466 | 466 |
| (ZmTERF20) | ZmTERF20-R | TTGCTGGAATCACTTCATGG |  |  |
| GRMZM2G161146 | ZmTERF21-F | AAAACGTCTCTCGCCGTCT | 1191 | 1191 |
| (ZmTERF21) | ZmTERF21-R | GCTGCTAATGCGCCTAATCT |  |  |
| GRMZM2G012999 | ZmTERF22-F | AATTCACCATCCAACGGACT | 1247 | 1247 |
| (ZmTERF22) | ZmTERF22-R | TGGGCTTCAGTCATTCAGTG |  |  |
| -^c^ | ZmTERF23-F | ACTCAGAGCTCCAATGGCC | ?^e^ | 1868 |
| (ZmTERF23*) | ZmTERF23-R | ACACGGTAAGGAAGCAATGC |  |  |
| GRMZM2G142150 | ZmTERF24-F | GGCTTCCTCCACCCTCTAAT | 3728 | 2105 |
| (ZmTERF24) | ZmTERF24-R | AACAGCCTCACCAATCCAAG |  |  |
| GRMZM2G062910 | ZmTERF25-F | CCCTTCACTTCTCGCTCATC | 1871 | 1871 |
| (ZmTERF25) | ZmTERF25-R | AAAGGATGCACATGGCAAAT |  |  |
| GRMZM2G325350 | ZmTERF26-F | TTGAGGTGTTCGTGGTGCTA | 1338 | 1338 |
| (ZmTERF26) | ZmTERF26-R | ATGGCGCCTAAACTTGTGAC |  |  |
| GRMZM2G029933 | ZmTERF27-F | CCCCAAGTCCAAATGATGAA | 1552 | 1552 |
| (ZmTERF27) | ZmTERF27-R | TTCGCGTACCAAGCACATAC |  |  |
| GRMZM2G068462 | ZmTERF28-F | CATCTCTGTCGCATTCTCCA | 594 | 594 |
| (ZmTERF28) | ZmTERF28-R | CACGCCGTTATATTTCCGTTA |  |  |
| GRMZM2G157716 | ZmTERF29-F | GCACGAGGGCTTGGATTATG | 971 | 971 |
| (ZmTERF29) | ZmTERF29-R | AGCACGCAACCAGATTCATC |  |  |

^a^ Putative gene model annotated in MaizeGDB ([http://www.maizegdb.org](http://www.maizegdb.org/)) for corresponding *ZmTERF* gene in parentheses was confirmed via PCR amplication.

^b^ The theoretical length of PCR amplicons in base pairs (bp) for the *ZmTERF* gene models with B73 seedling DNA and cDNA as templates, respectively.

^c^ The minus (-) denotes that there is no correct gene models for *ZmTERF23** gene wich is predicted from two adjacent gene models, *GRMZM2G426154* and *GRMZM2G175930* (Figure S1).

^d^ There were two transcripts for *ZmTERFs* identified by PCR and DNA sequencing.

^e^ Length of *ZmTERF23* DNA sequence could not be determined because of the gap within maize genome.
