# Supplementary material for: Genome-Wide Identification, Evolution and Expression Analysis of mTERF Gene Family in Maize
Source: PLoS One. 2014 Apr 9;9(4):e94126. doi: 10.1371/journal.pone.0094126 (PMC3981765; doi:10.1371/journal.pone.0094126)
Supplement: Table S7 — Digital expression analysis of maize mTERF genes. (DOC) [file pone.0094126.s014.doc]

**Table S7. Digital expression analysis of maize mTERF** genes.

| Gene | Tissue and organ type^a^ | | | | | | |
| --- | --- | --- | --- | --- | --- | --- | --- |
|  | Root | Shoot | Ear | Embryo | Endosperm | Tassel | Multiple |
| ZmTERF1 |  |  |  | + |  |  | + |
| ZmTERF2 | + | + | + |  |  |  | + |
| ZmTERF3 |  | + |  | + |  |  | + |
| ZmTERF4 |  |  |  |  |  |  |  |
| ZmTERF5 | + | + | + | + | + |  | + |
| ZmTERF6 | + | + | + | + | + | + | + |
| ZmTERF7 |  |  |  |  |  |  |  |
| ZmTERF8 | + | + | + | + | + | + | + |
| ZmTERF9 |  |  | + |  |  |  | + |
| ZmTERF10 | + | + |  |  | + | + | + |
| ZmTERF11 |  | + |  |  |  |  | + |
| ZmTERF12 |  | + |  | + |  |  | + |
| ZmTERF13 |  |  |  |  |  |  | + |
| ZmTERF14 |  |  |  |  |  |  | + |
| ZmTERF15 |  |  | + |  | + |  | + |
| ZmTERF16 |  |  | + |  | + |  | + |
| ZmTERF17 |  | + |  | + |  |  | + |
| ZmTERF18 |  |  |  |  |  |  | + |
| ZmTERF19 |  |  |  |  |  |  | + |
| ZmTERF20 |  |  |  |  |  |  |  |
| ZmTERF21 |  | + |  |  |  |  | + |
| ZmTERF22 |  |  | + |  |  | + | + |
| ZmTERF23 |  |  |  |  |  |  | + |
| ZmTERF24 |  | + |  | + |  |  | + |
| ZmTERF25 |  |  | + |  | + |  | + |
| ZmTERF26 |  | + |  | + |  |  | + |
| ZmTERF27 | + | + |  | + |  |  | + |
| ZmTERF28 |  |  |  |  |  |  | + |
| ZmTERF29 |  | + |  |  |  |  | + |
| Toral | 6 | 14 | 9 | 10 | 7 | 4 | 26 |

^a^ Transcripts of *ZmTERF* genes were used to search maize dbEST data in NCBI via blastn (<http://blast.ncbi.nlm.nih.gov/Blast.cgi>) and the hits with the cutoff sets, length >200bp, maximum identity > 95% and an E-value < 10^-10^ were collected and denoted by a plus (+).
