# Supplementary material for: Genome-Wide Identification, Evolution and Expression Analysis of mTERF Gene Family in Maize
Source: PLoS One. 2014 Apr 9;9(4):e94126. doi: 10.1371/journal.pone.0094126 (PMC3981765; doi:10.1371/journal.pone.0094126)
Supplement: Table S9 — Gene-specific primers used for qRT-PCR analysis of maize mTERF genes. (DOC) [file pone.0094126.s016.doc]

**Table S9. Gene-specific primers used for qRT-PCR analysis of maize mTERF** genes.

| Gene | Forward Primer (5'->3') | Reverse Primer (5'->3') | Amplicon (bp) |
| --- | --- | --- | --- |
| ZmTERF2 | ACGACATGAACAAGGCGTTG | TTGATCATGTGCGACACGTC | 115 |
| ZmTERF5 | AACTTGGGCATCTTCAACGC | AATTCCTTCACGCGTTCGAG | 146 |
| ZmTERF6 | AGGAGAGGGAGAAGGAGGTT | ATTCCCCAGTCACTGTCCAG | 184 |
| ZmTERF8 | TTTCCAAGCTGCCATTGGTG | AGGCCTGTGAGCAATGTACTC | 110 |
| ZmTERF9 | CTGCTGCTCCTTCGATGTTC | GAATCTGTGGGCTTAGCTGC | 129 |
| ZmTERF11 | TTTTGTTCAGGCACGCTGTG | TTTGCACCATTCTGGCAACC | 123 |
| ZmTERF12 | AAACATTGCCTGGAGCCAAG | GCTCGAGTTCATCTGCTTAAGC | 150 |
| ZmTERF13 | TCCCGCACTACTTTGCATTC | CCCTGAAATCGTCATCGTTGC | 123 |
| ZmTERF15 | TGTTGCCACGGAATTGCTTG | TCGTAAGGATGCACGTATCGG | 127 |
| ZmTERF19 | ACAGGTGCTTCTTCAACGTG | TCATAAGCATCGGCAAGACC | 103 |
| ZmTERF21 | TTCATGGGTTGCTTCGATCG | AAACAGTTAGACCGCACTGC | 124 |
| ZmTERF23 | ATGAAGAGTTTGCGCAGAGG | TTTTGGCATGCTGCTGCTTC | 145 |
| ZmTERF24 | TCAGATGGGAGGCCACTTTT | CGATACAGGAACCAAGCAGC | 161 |
| ZmTERF26 | ACAGCACCAAGGATTTGCTG | TCGGAAAAACGCGCAAGATC | 96 |
| ZmTERF27 | AAAGCCTTTCGTCGAACACC | TGTAGCCCAAATCCAAGCAC | 99 |
| ZmTERF28 | TCGCAGGTGAAGGACAAGATC | GACTGGAGGAACGACACCAC | 131 |
| ZmTERF29 | GAGTTCCTCGAGTCGCTAGG | GAAGTACTCGGGGAACTCGT | 177 |
| Actin1^a^ | TTTAAGGCTGCTGTACTGCTGTAGA | CACTTTCTGCTCATGGTTTAAGG | 120 |

^a^ Maize *Actin1* gene (Genebank #J01238) is used as an internal control [74].
